# Acss2/HIF-2 signaling facilitates colon cancer growth and metastasis

**DOI:** 10.1371/journal.pone.0282223

**Published:** 2023-03-02

**Authors:** Joseph A. Garcia, Rui Chen, Min Xu, Sarah A. Comerford, Robert E. Hammer, Shelby D. Melton, Linda A. Feagins

**Affiliations:** 1 Department of Medicine, Columbia University Medical Center, New York, New York, United States of America; 2 Research & Development, James J. Peters Veterans Affairs Medical Center, New York, New York, United States of America; 3 Department of Medicine, University of Texas Southwestern Medical Center, Dallas, Texas, United States of America; 4 Department of Molecular Genetics, University of Texas Southwestern Medical Center, Dallas, Texas, United States of America; 5 Department of Biochemistry, University of Texas Southwestern Medical Center, Dallas, Texas, United States of America; 6 Pathology & Laboratory Medicine, Veterans Affairs North Texas Health Care System, Dallas, Texas, United States of America; 7 Department of Pathology, University of Texas Southwestern Medical Center, Dallas, Texas, United States of America; 8 Department of Internal Medicine, Dell Medical School, The University of Texas at Austin, Austin, Texas, United States of America; University of Kentucky, UNITED STATES

## Abstract

The microenvironment of solid tumors is characterized by oxygen and glucose deprivation. Acss2/HIF-2 signaling coordinates essential genetic regulators including acetate-dependent acetyl CoA synthetase 2 (Acss2), Creb binding protein (Cbp), Sirtuin 1 (Sirt1), and Hypoxia Inducible Factor 2α (HIF-2α). We previously shown in mice that exogenous acetate augments growth and metastasis of flank tumors derived from fibrosarcoma-derived HT1080 cells in an Acss2/HIF-2 dependent manner. Colonic epithelial cells are exposed to the highest acetate levels in the body. We reasoned that colon cancer cells, like fibrosarcoma cells, may respond to acetate in a pro-growth manner. In this study, we examine the role of Acss2/HIF-2 signaling in colon cancer. We find that Acss2/HIF-2 signaling is activated by oxygen or glucose deprivation in two human colon cancer-derived cell lines, HCT116 and HT29, and is crucial for colony formation, migration, and invasion in cell culture studies. Flank tumors derived from HCT116 and HT29 cells exhibit augmented growth in mice when supplemented with exogenous acetate in an Acss2/HIF-2 dependent manner. Finally, Acss2 in human colon cancer samples is most frequently localized in the nucleus, consistent with it having a signaling role. Targeted inhibition of Acss2/HIF-2 signaling may have synergistic effects for some colon cancer patients.

## Introduction

To survive in hostile environments, organisms must sense environmental threats and respond with self-protective mechanisms that include immediate as well as delayed molecular, biochemical, and cellular responses. Several protective actions result from transcriptional events induced when specific genetic regulators are activated by stress. Some genetic regulators may directly sense and respond to changes in intracellular metabolism induced by an environmental stress. Alternatively, signal transducers may be affected by changes in levels of extracellular metabolites that are transported into the cell through active or passive mechanisms.

The Hypoxia Inducible Factor (HIF) transcription factor family in metazoans is comprised of three regulated alpha subunits and one beta subunit [1]. HIFs respond to hypoxia, but are also activated by other environmental stresses. For example, HIF-2 [2], but not HIF-1 [3], is activated by glucose deprivation. Furthermore, although HIF-1α is largely controlled by oxygen-dependent prolyl hydroxylases (PHDs) and an asparaginyl hydroxylase (Factor Inhibiting HIF-1, FIH1) [4–6] that regulate protein degradation and coactivator recruitment, respectively, maximal HIF-2 signaling also requires cyclical acetylation and deacetylation of HIF-2α [7–9]. Given their prominent role in environmental stress response, HIF members play a prominent role in cancers where rapid cellular proliferation profoundly affects local oxygen and glucose availability [10].

Although the initial focus was on its role as a cytosolic enzyme [11], the acetate-dependent acetyl CoA generator Acss2 has subcellular compartment-specific roles [12]. In the cytosol, Acss2 provides acetyl CoA for lipid biosynthesis and other anabolic processes. In response to physiological and pathophysiological stress [13–16], Acss2 is enriched in or translocates into the nucleus in normal tissues, where it likely regulates *de novo* genetic and epigenetic events. We have shown that Acss2 augments stress signaling by HIF-2 in fibrosarcoma (HT1080) and hepatocarcinoma (Hep3B) derived cell lines subjected to oxygen or glucose deprivation [15, 16]. HT1080 cancer cells exposed to oxygen or glucose deprivation generate acetate, which stimulates Acss2 nuclear translocation [14, 15]. Nuclear Acss2 is required for acetylation of HIF-2α by Cbp and stable Cbp/HIF-2α complex formation in HT1080 cells. Flank tumors derived from HT1080 cells are dependent upon nuclear-localizing Acss2 as well as acetylated HIF-2 for maximal growth. Supplementing mice with exogenous acetate augments HT1080 cell tumor growth and metastasis in an Acss2 and HIF-2 dependent manner.

In this report, we assess the role of Acss2/HIF-2 signaling in colon cancer using cell, mouse, and human colon cancer studies. We examine molecular features of Acss2/HIF-2 signaling in HCT116 and HT29 colon cancer cells exposed to oxygen or glucose deprivation. We assess whether colony formation, migration, and invasion of HCT116 and HT29 colon cancer cell lines are regulated by Acss2/HIF-2 signaling. We measure growth and metastasis of HCT116 and HT29 derived flank tumors with or without an intact Acss2/HIF-2 signaling axis in mice receiving vehicle or exogenous acetate. Finally, we examine the pattern as well as presence of Acss2 immunoreactivity in the cytosol and nucleus of human colon cancer samples compared to benign tissue obtained from the same patient.

## Materials and methods

### Cell culture

We maintained HCT116 cells (Cat. No. CCL-247, ATCC, Manassas, VA), HT29 cells (Cat. No. HTB-38, ATCC), and HEK293T cells (Cat. No. CRL-3216, ATCC) as previously described with the following modifications [7]. Cells were grown in either complete (high glucose) medium [Dulbecco’s Modification of Eagle’s Medium (DMEM) with 4.5 g/L (25 mM) glucose, L-glutamine, sodium pyruvate (Cat. No. 10-013-CV, Corning Cellgro, Manassas, VA); 10% heat-inactivated fetal bovine serum (FBS; Cat. No. F4135, Sigma, Saint Louis, MO); 1% penicillin/streptomycin (Cat. No. 30-002-CI, Corning Cellgro)] or in low glucose medium [DMEM with L-glutamine (Cat. No. 11966–025, Gibco, Life Technologies, Grand Island, NY) supplemented with glucose to final concentration of 1 mM; 10% heat-inactivated FBS; 1% penicillin/streptomycin]. For hypoxia treatments, cells were incubated in either a standard incubator (5% CO_2_, 21% O_2_) or were housed in an incubator located within the hypoxia workstation (5% CO_2_, 1% O_2_). For hypoxia samples, we prepared extracts within a hypoxia workstation (Coy Laboratories, Grass Lake, MI). For low glucose and acetate treatments, we prepared extracts under normal oxygen conditions. For short chain fatty acid (SCFA) addition, we added sterile acetate to complete medium for a final concentration of 5 mM.

### Acetate determinations

For hypoxia treatment, we transferred 80% confluent HCT116 or HT29 cells grown under standard growth conditions to an incubator within the hypoxia workstation after changing the standard medium. For low glucose treatment, we maintained the cells within the standard incubator after changing to low glucose medium. At the time of harvest, we aspirated off medium, quickly added 1 ml of ice-cold 0.1 N HCl, and scraped cells with a spatula. After transferring to an ice-cold microfuge tube, cellular debris was pelleted at 14,000 rpm, 4°C, 10 min. We transferred 0.9 ml supernatant to a new, ice-cold microfuge tube, and adjusted the pH to 6.5–7.0 with 5 N NaOH. Extracts were stored at -80°C until assay with the Megazyme Acetic Acid Rapid Kit (Cat. No. K-ACETRM, Megazyme, Ireland) according to the manufacturer’s protocol [16].

### Immunodetection

Extract preparation, immunoblotting, and detection by film were performed as previously described [14, 15]. Proteins were immunoblotted using the following antibodies: human p300 (1:500 dilution; Cat. No. sc-584, Santa Cruz Biotechnology, Santa Cruz, CA), human CBP (1:500 dilution; Cat. No. 4772, Cell Signaling Technology, Danvers, MA), human Acss2 (1:500 dilution; Cat. No. ab66038, Abcam, Cambridge, MA), human HIF-2α (1:1,000 dilution; Cat. No. NB100-132, Novus Biologicals, Littleton, CO), α-tubulin (1:10,000 dilution; Cat. No. T9026, Sigma).

### HIF-2α acetylation determination

HCT116 or HT29 cells grown in a single 100 mm plate were pretreated for 6 hr in complete medium with 5 μM sirtinol (Cat. No. 510 8474, Chembridge Corporation, San Diego, CA) and 10 mM nicotinamide (NAM; Cat. No. N0636, Sigma, St. Louis, MO), and then cultured in the appropriate medium under the indicated conditions. Extracts were prepared using a kit (Cat. No. 40010, Active Motif) supplemented with 1× protease inhibitor cocktail (Cat. No. P8340, Sigma, St. Louis, MO), 1 mM PMSF (phenylmethylsulfonyl fluoride, Cat. No. P7626, Sigma), 10 mM NAM, and 5 μM sirtinol. Extracts containing HIF-2α were mixed with a monoclonal human HIF-2α antibody (Cat. No. NB100-132, Novus Biologicals) for 1 hr, then immunoprecipitated using protein G magnetic beads (Cat. No. 54002, Active Motif, Carlsbad, CA). Aliquots were immunoblotted for HIF-2α or acetyl lysine as previously described [14, 15].

### Endogenous protein immunoprecipitation

Immunoprecipitation of endogenous proteins was accomplished using a Universal Co-IP kit (Cat. No. 54002, Active Motif). HCT116 or HT29 nuclear extracts were first cleared with protein A agarose beads, then incubated with HIF-2α antibody (Cat. No. NB100-132, Novus Biologicals, Littleton, CO) or normal mouse IgG (Cat. No. sc-2025, Santa Cruz Biotechnology, Santa Cruz, CA) for 2 hr before addition of protein A agarose beads. After binding, beads were pelleted by centrifugation and washed with buffer, then immunoprecipitated materials were eluted and immunoblotted with anti-human p300 (1:500 dilution; Cat. No. sc-584, Santa Cruz Biotechnology, Santa Cruz, CA), anti-human CBP (1:500 dilution; Cat. No. 4772, Cell Signaling Technology, Danvers, MA), or anti-HIF-2α (1:1,000 dilution; Cat. No. NB100-132, Novus Biologicals) primary antibodies.

### Lentiviral expression and reporter plasmid studies

Wild-type (WT) human HIF-2α cDNA with intact lysine residues (K3) or a mutant cDNA with arginine substitutions for acetylated lysine residues (R3: K385R, K685R, K741R) containing a carboxy terminal hemagglutinin A (HA) tag were previously described [7]. Wild-type mouse Acss2 cDNA or a cytosol-restricted mutant cDNA (ED: R668E, K669D) containing an amino terminal V5 epitope tag were previously described [7, 14, 15]. The HIF-2α and Acss2 cDNAs contained missense mutations to render them resistant to shRNA targeting the human mRNAs. The control expression cDNA used in the LTV knockdown/rescue experiments was a mouse Acss2 cDNA with deletions of sequences corresponding to coding exons 3 through 6, which encodes a truncated and non-functional Acss2 protein generated in the mouse Acss2 knockout [16].

We used pLenti6 derivatives (Invitrogen, Life Technologies, Grand Island, NY) to generate lentivirus (LTV) containing CMV promoter-driven expression vectors that harbor the indicated human HIF-2α or mouse Acss2 cDNA in place of the parental lacZ-encoding cDNA. The expression cassette was linked to a reporter (DsRed for cell studies, firefly luciferase for mouse flank tumor studies) by an internal ribosome entry site (IRES), which was followed by a concatamer of four different shRNAs for the indicated gene or by a tandem pair of two control shRNAs for efficient shRNA-mediated knockdown [17, 18].

Lentiviruses were generated by co-transfection of the expression vector of interest with packaging plasmids psPAX2 and pMD2G into HEK293T cells as previously described [14, 15]. The day before transduction, HCT116 or HT29 cells were trypsinized and 2 x 10^5^ cells per well plated in 1 mL complete culture medium in a 6-well plate for overnight incubation at 37°C. On the day of transduction, medium was removed and replaced with 1 ml of complete medium with 10 μg/ml polybrene (Cat. No. 107689, Sigma, St. Louis, MO). Lentiviral particles were thawed to room temperature, mixed gently, and added to the HCT116 or HT29 cells. After gently swirling to mix, cells were incubated overnight. After 12 hr, culture medium was replaced with 2 ml of complete medium containing 10 μg/ml blasticidin S (Cat. No. ant-bl, Invivogen, San Diego, CA), which was replaced every 2 days until one week after all control cells had died. Positive cells were propagated in 1 μg/ml blasticidin S for two weeks, and then were frozen down. For experiments, cells were thawed and allowed to grow for three passages without drug selection before use.

### Real-time PCR analyses

Expression of *VEGFa*, *PAI1*, *MMP9*, *GLUT1*, *PGK1*, and *PPIB* were determined by reverse transcription of total RNA followed by semi-quantitative real-time PCR analysis on an Applied Biosystems ABI Prism 7000 thermocycler using Power SYBR Green Master Mix following the manufacturer’s protocol as previously described [7, 14, 15]. We determined the relative levels of gene expression from a single pooled sample made from three individually wells (biological replicates) from a 12-well plate for stable knockdown HCT116 or HT29 cells maintained under the indicated condition. The results of triplicate experiments, with each sample measured as triplicates, were expressed as 2 ^-(gene-of-interest number of cycles- cyclophilin number of cycles)^.

### Colony formation assays

For colony formation assays, 5 x 10^2^ HCT116 or HT29 cells seeded in triplicate 10 cm plates were allowed to attach for 24 hr in complete medium. After 24 hr, medium was changed and cells were cultured for 10 days under control oxygen (21% O_2_) and glucose (25 mM) conditions, hypoxia (1% O_2_), low glucose (1 mM), or acetate supplemented (complete medium with 5 mM acetate) conditions. Medium was not changed throughout the experiment. Colonies were stained with 1% crystal violet in ethanol/PBS (15%/85%). Cells were imaged and colony number determined using ImageJ software.

### Cell migration and cell invasion assays

For cell migration assays, HCT116 or HT29 cells were serum-starved in 0.5% FBS/DMEM medium overnight. After 12 hr, 1.5 x 10^5^ HCT116 or HT29 cells in serum-free medium were transferred into a transwell insert. For cells maintained under normal conditions, cells were incubated with complete medium and exposed to 21% oxygen for 4 hr. For cells maintained under hypoxic conditions, cells were incubated with complete medium and exposed to 1% oxygen for 4 hr. For cells exposed to low glucose conditions, cells were incubated with low glucose (1 mM) medium at 21% oxygen for 24 hr and compared to control cells maintained under standard glucose (25 mM) conditions for 24 hr. For cells supplemented with acetate, cells were incubated with complete medium supplemented with acetate (5 mM) and exposed to 21% oxygen for 4 hr. Cell migration was assessed in triplicates for each treatment after crystal violet staining. The absorbance was recorded at 560 nm with a microplate reader.

For cell invasion assays, we used a kit containing Matrigel pre-filled wells (CytoSelect 24-Well Cell Invasion Assay Kit; Cat. No. CBA-110, Cell Biolabs, San Diego, CA). HCT116 or HT29 cells were serum-starved overnight in 0.5% FBS/DMEM medium. After pre-incubating the transwell insert for 1 hr with serum-free medium at room temperature, 1.5 x 10^5^ HCT116 or HT29 cells in serum-free medium were transferred into the transwell insert. After 12 hr for each treatment, cell migration was determined in triplicates according to the manufacturer’s protocol.

### *In vivo* nude mice flank tumor humane endpoints

All animal experiments were approved by the University of Texas Southwestern Medical Center Institutional Animal Care and Use Committee, APN 2013–0098. Mice were weighed prior to tumor cell injections and were monitored daily for signs of distress beginning three days after tumor cell implantation defined as increased respiratory effort, weight loss >20% from initial weight, poor grooming or porphyrin tears, reduced spontaneous movement, extreme lethargy, gait abnormalities, tumor ulceration, apparent sickness or overt anatomical abnormalities that precluded adequate food and water intake. Mice considered distressed were euthanatized immediately. If not in apparent distress, mice were euthanatized within 24 hr if a tumor reached a pre-specified size defined as estimated tumor mass greater than 10% of body weight (assuming 1 cm^3^ equals 1 gm), estimated tumor volume reached ~1.5 cm^3^, or if any tumor dimension exceeded more than 1.5 cm. Mice were euthanized under isoflurane anesthesia by bilateral thoracotomy, consistent with guidelines of the American Veterinary Medical Association Panel on Euthanasia.

### *In vivo* nude mice flank tumor experiments

Female nude mice obtained from NCI were anesthetized by isoflurane inhalation and then injected subcutaneously on the left dorsal flank with 5×10^6^ luciferase-expressing stably transformed HCT116 or HT29 cells grown using 3% FBS to increase subsequent *in vivo* tumor cell growth and seeding efficiency and suspended in 0.5 ml DMEM. Tumor mass assessments were made by caliper measurements performed daily starting three days after cell injections. Starting six days after injection, mice were administered vehicle (PBS, 0.01 mL/g body weight) or the short chain fatty acid (SCFA) compounds glyceryl triacetate (GTA, triacetin; 90 μL/25 gm body weight; Cat. No. W200700, Sigma-Aldrich Chemicals, Saint Louis, MO), glyceryl tributyrate (GTB, tributyrin; 140 μL/25 g body weight; Cat. No. W222305, Sigma) or glyceryl tripropionate (GTP, tripropionin; 115 μL/25 g body weight; Cat. No. W328618, Sigma) by oral gavage once per day using a 20-gauge stainless steel animal feeding tube (Cat. No. FTSS-20S-25, Instech Laboratories, Inc., Plymouth Meeting, PA) [19]. The experiment was considered completed when tumor volumes reached ~1.5 cm^3^ or if any tumor dimension exceeded 1.5 cm for at least 25% of mice in the largest tumor-bearing set. No mice were euthanatized for distress. For the flank tumor experiments using wildtype HCT116 and HT29 cells, 6 mice per group were injected with cells. No mice died or were euthanatized for distress in these sets. The experiment was concluded 10 days after the initial injection of tumor cells, which was when the acetate-treated (GTA) group had achieved the pre-specified humane endpoint for maximal tumor size with both wildtype HCT116 and HT29 cells. For the flank tumor experiments using Acss2 or HIF-2 knockdown/rescue HCT116 and HT29 cell lines, 7 mice per group were injected with cells. No mice were euthanatized for distress in these sets. A flank tumor was not visible for one mouse in the triacetin-treated mutant Acss2 rescue HCT116 cell line group and this mouse was eliminated from analysis. One mouse in the triacetin-treated wildtype HIF-2 rescue HCT116 cell line group was found dead on day 5 and this mouse was eliminated from analysis. There was no obvious cause of death for this mouse and the tumor size on the day prior to death was fourth in size for the seven mice in this group. All experiments using Acss2 or HIF-2 knockdown/rescue HCT116 and HT29 cell lines were concluded 10 days after the initial injection of tumor cells, which was when the acetate-treated (GTA) group had achieved the pre-specified humane endpoint for maximal tumor size for either the WT HIF-2 knockdown/rescue (HCT116) or WT Acss2 knockdown/rescue (HT29) group. No fewer than 6 mice were obtained for all groups in the flank tumor studies.

### *Ex vivo* nude mice flank tumor experiments

After mice were sacrificed, primary tumor, lung, and liver were removed for biochemical luciferase activity determination as previously described [14, 15]. Individual tissues were weighed as were smaller samples. The latter were homogenized in lysis reagent (25 mM Tris-phosphate pH 7.8, 2 mM DTT, 2mM 1,2 diaminocyclohexane-N,N,N,N-tetra-acetic acid, 10% glycerol, 1% NP-40) containing soybean trypsin inhibitor (0.2 mg/ml) and bovine serum albumin (0.2 mg/ml). Duplicate samples (2 μl tumor, 20 μl lung or liver lysates) were diluted in 100 μl lysis reagent containing 2.5 mM MgCl_2_. Immediately prior to measurement, 50 μl luciferin reagent (20 mM tricine, 1 mM (MgCO_3_)_4_Mg(OH)_2_^.^5H_2_O, 2.67 mM MgSO_4_, 0.1 mM EDTA, 33 mM DTT, 0.27 mM coenzyme Q, 0.47 mM luciferin, 0.53 mM ATP, pH 7.8) was added. Measurements were made for 10 sec in a single-tube luminometer (Sirius, Berthold Detection Systems, Pforzheim, Germany). Tumor cell burden for each tissue was assessed by multiplying the luciferase activity for the respective sample by total tissue weight.

### Immunohistochemistry of human colon cancer samples

We performed immunohistochemistry (IHC) on formalin-fixed and paraffin-embedded 5 μm sections from 34 paired stage I-IV human colon cancer and benign tissue samples mounted on poly-l-lysine–coated glass slides and collected by the University of Texas Southwestern Medical Center Tissue Management Core. Tumor and benign tissue samples were present on the same slide when undergoing IHC. We baked slides at 56°C for 30 min, deparaffinized in xylene, and then rehydrated through graded alcohols. We quenched endogenous peroxidase activity by incubating slides in 3% hydrogen peroxide (Cat. No. H1009-100ML, Sigma, Saint Louis, MO) in deionized water for 30 min and rinsing in deionized water twice for 5 min. For Acss2 staining, we submerged slides in BD Retrievagen A (pH 6.0) working solution (Cat. No. 550524, BD Biosciences, San Jose, CA) and heated to ~90°C in a microwave oven for 10 min, followed by slow cooling to room temperature (RT) over 60 min. We then rinsed slides twice with deionized water and once with PBS for 10 min. We blocked non-specific binding by a 60 min incubation with 10% goat serum (Cat. No. S-1000, Vector Laboratories, Burlingame, CA) in PBS. Next, we incubated slides overnight at 4°C with a rabbit monoclonal antibody directed against Acss2 (Cat. No. 3658, Cell Signaling Technology, Danvers, MA; 1:150 in 10% goat serum with PBS) and then 2 hr with biotin-conjugated goat anti-rabbit IgG (Cat. No. 111-065-045, Jackson ImmunoResearch Laboratories, Inc., West Grove, PA; 1:500 in 10% goat serum with PBS). After rinsing in PBS, we incubated slides with HRP-conjugated streptavadin (Cat. No. 43–4323, Invitrogen Corporation, Camarillo, CA; 1:750 in 10% goat serum with PBS) for 60 min at RT. After rinsing in PBS, we visualized immunoreactive deposits by incubation with 3′-amino-9-ethylcarbazole (Cat. No. 00–2007, Invitrogen Corporation, Camarillo, CA). Finally, we counterstained slides with hematoxylin QS nuclear counterstain (Cat. No. H-3404, Vector Laboratories, Burlingame, CA) for 2 min and then coverslipped using Aqua-Poly/Mount Coverslipping Medium (Cat. No. 18606–20, Polysciences, Inc., Warrington, PA).

### Immunoreactivity scoring of human colon cancer samples

Qualified investigators blinded to tumor diagnosis, stage, TNM stage, and grade evaluated colon cancer and benign samples for Acss2 immunohistochemistry (IHC). The entire slide for each sample was first evaluated individually, then scores were compared and discussed to arrive at a unique determination for each tumor/normal tissue pair. Scores of randomly selected samples were adjudicated by a second team in a similar manner to confirm scoring. A semi-quantitative scoring system was used to gauge Acss2 levels and pattern in two subcellular locations, cytosol and nucleus. The intensity of staining was gauged as follows: 0 = none, 1 = faint, 2 = mild, 3 = moderate, 4 = marked, with the highest number present being recorded. A sum of intensity was made for cytosolic and nuclear locations in both tumor and benign tissues, which was used to calculate relative ratios of cytosolic/nuclear Acss2 IHC intensity. The pattern of IHC staining was also gauged as follows: N = none, D = diffuse, L = localized. The number of samples with Acss2 IHC patterns designated none, diffuse, localized, and present (diffuse or localized) was noted for cytosolic and nuclear locations in both tumor and benign tissues. In addition, a select low power (10x) image was obtained to provide a “representative image” for each benign and tumor slide. Scoring data was tabulated for the entire slide as well as for the select images using criteria as outlined above.

### Statistical analyses

We performed one-tailed or two-tailed analyses by unpaired Student’s t-test with Welch’s correction or by Z-test for groups of equal or unequal sample size, respectively. We assumed equal variances for experimental groups. We used one-way or two-way ANOVA analyses for multiple comparisons with Dunnett’s multiple comparison post-hoc test. The statistical analyses were performed using Excel, StatPlus (AnalystSoft Inc.), or Prism 7 (GraphPad Software, Inc.). All P values less than or equal to 0.05 (*) are noted for the stated comparisons.

## Results

### Stress induces Cbp/HIF-2α interactions in colon cancer cells

**Fig 1:**
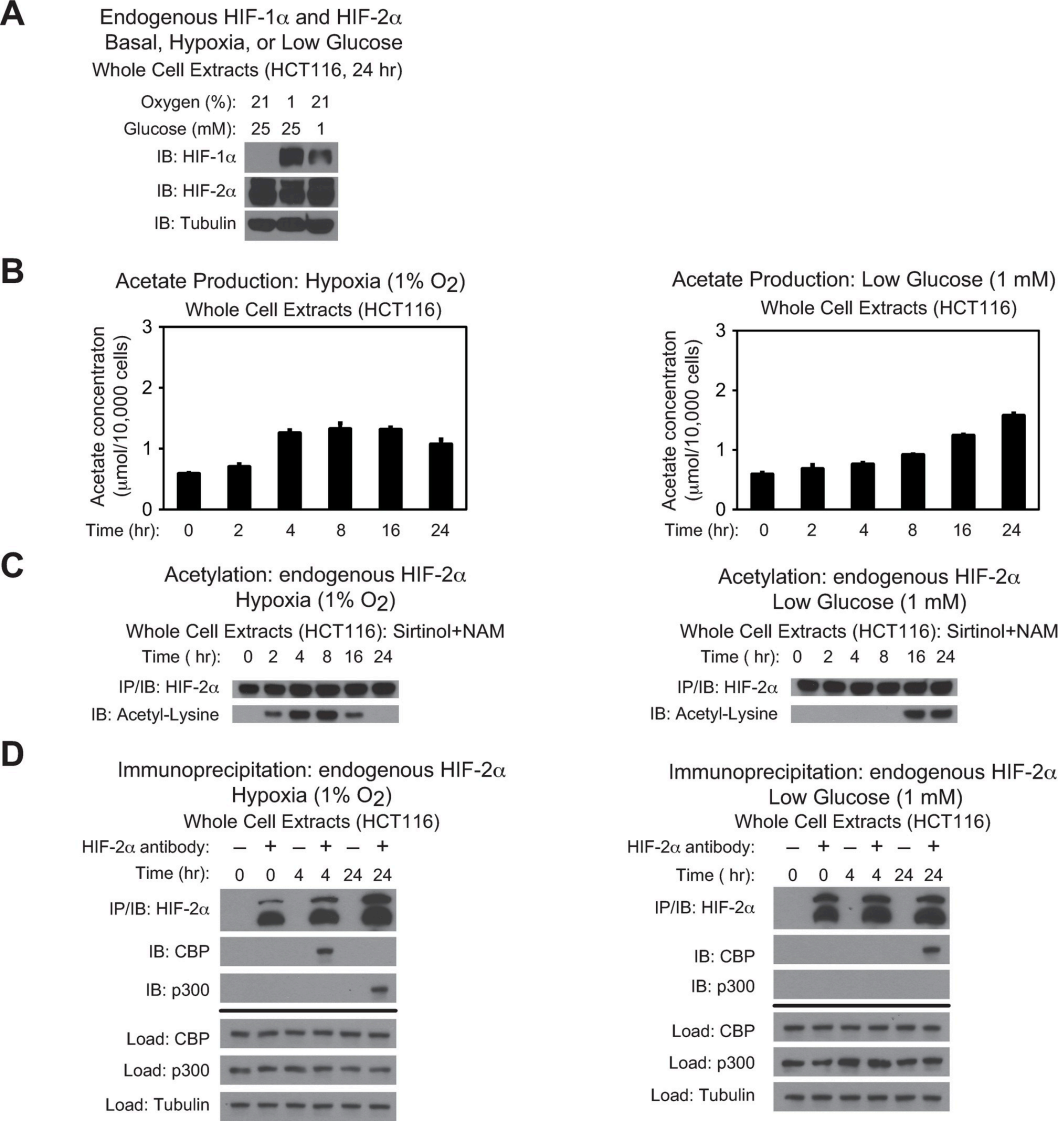
Stress induces CBP/HIF-2α interactions in HCT116 cells. (A) Detection of endogenous HIF-1α or HIF-2α in HCT116 whole cell extracts by immunoblotting after the indicated period of hypoxia exposure or glucose deprivation. Alpha-tubulin levels for each immunoblot are also shown. (B) Cellular acetate levels generated in HCT116 cells after the indicated period of hypoxia exposure (left side) or glucose deprivation (right side) (*n* = 3 biological replicates/time-point; single measurement/replicate; mean/SD) differed as a function of time (P<0.001, one-way ANOVA). (C) Acetylation of endogenous HIF-2α in HCT116 cells after immunoprecipitation (IP) and detection by immunoblotting (IB) after the indicated period of hypoxia exposure (left side) or glucose deprivation (right side) with pharmacological inhibition of Sirt1 by sirtinol and nicotinamide (NAM). (D) Detection of endogenous CBP/HIF-2α or p300/HIF-2α complexes in HCT116 cells by immunoblotting (IB) after early (4 hr) and late (16 hr) hypoxia exposure (left side), or after early (2 hr) and late (24 hr) low glucose exposure (right side).

**Fig 2:**
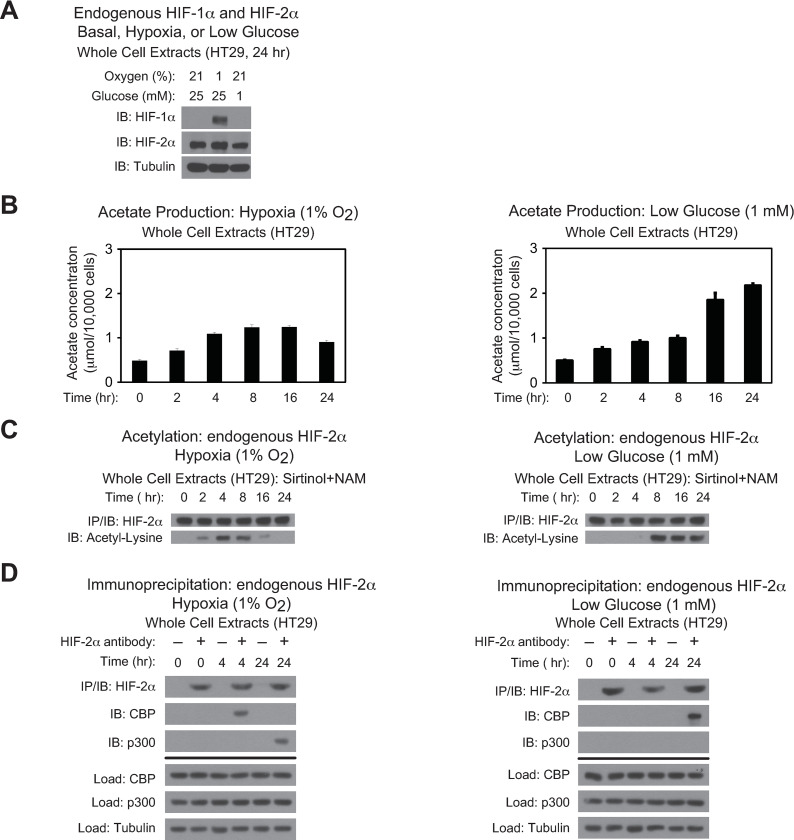
Stress induces CBP/HIF-2α interactions in HT29 cells. (A) Detection of endogenous HIF-1α or HIF-2α in HT-29 whole cell extracts by immunoblotting after the indicated period of hypoxia exposure or glucose deprivation. Alpha-tubulin levels for each immunoblot are also shown. (B) Cellular acetate levels generated in HT29 cells after the indicated period of hypoxia exposure (left side) or glucose deprivation (right side) (*n* = 3 biological replicates/time-point; single measurement/replicate; mean/SD) differed as a function of time (P<0.001, one-way ANOVA). (C) Acetylation of endogenous HIF-2α in HT29 cells after immunoprecipitation (IP) and detection by immunoblotting (IB) after the indicated period of hypoxia exposure (left side) or glucose deprivation (right side) with pharmacological inhibition of Sirt1 by sirtinol and nicotinamide (NAM). (D) Detection of endogenous CBP/HIF-2α or p300/HIF-2α complexes in HT29 cells by immunoblotting (IB) after early (4 hr) and late (16 hr) hypoxia exposure (left side), or after early (2 hr) and late (24 hr) low glucose exposure (right side).

To evaluate whether HIF-2 stress signaling is regulated by Acss2 in colon cancer derived cell lines, we first asked whether HIF-1α and HIF-2α were present in HCT116 and HT29 cell lines and whether their levels were affected by oxygen or glucose deprivation (Figs 1A and 2A). In both cell lines, HIF-1α protein was not detected under ambient oxygen and glucose levels, but was induced following hypoxia exposure. Glucose deprivation resulted in partial stabilization of HIF-1α protein in HCT116, but not in HT29 cells. In comparison, HIF-2α protein was readily detectable and did not vary significantly following oxygen or glucose deprivation in both cell lines. Similar to HT1080 cells, acetate levels in HCT116 and HT29 cell lines increased following oxygen or glucose deprivation with peak acetate levels at 4–8 hr during hypoxia exposure and by 24 hr after initiation of glucose deprivation (Figs 1B and 2B). Likewise, HIF-2α acetylation (Figs 1C and 2C) and Cbp/HIF-2α complex formation (Figs 1D and 2D) closely mirrored the rise in acetate in HCT116 and HT29 cell lines. As acetate levels returned to normal levels in late hypoxia, there was a decrease in HIF-2α acetylation. Moreover, p300/HIF-2α complex formation was only observed during late hypoxia, but was not evident under glucose deprivation in HCT116 and HT29 cells.

### Acss2/Cbp/HIF-2α interactions in colon cancer cells

**Fig 3:**
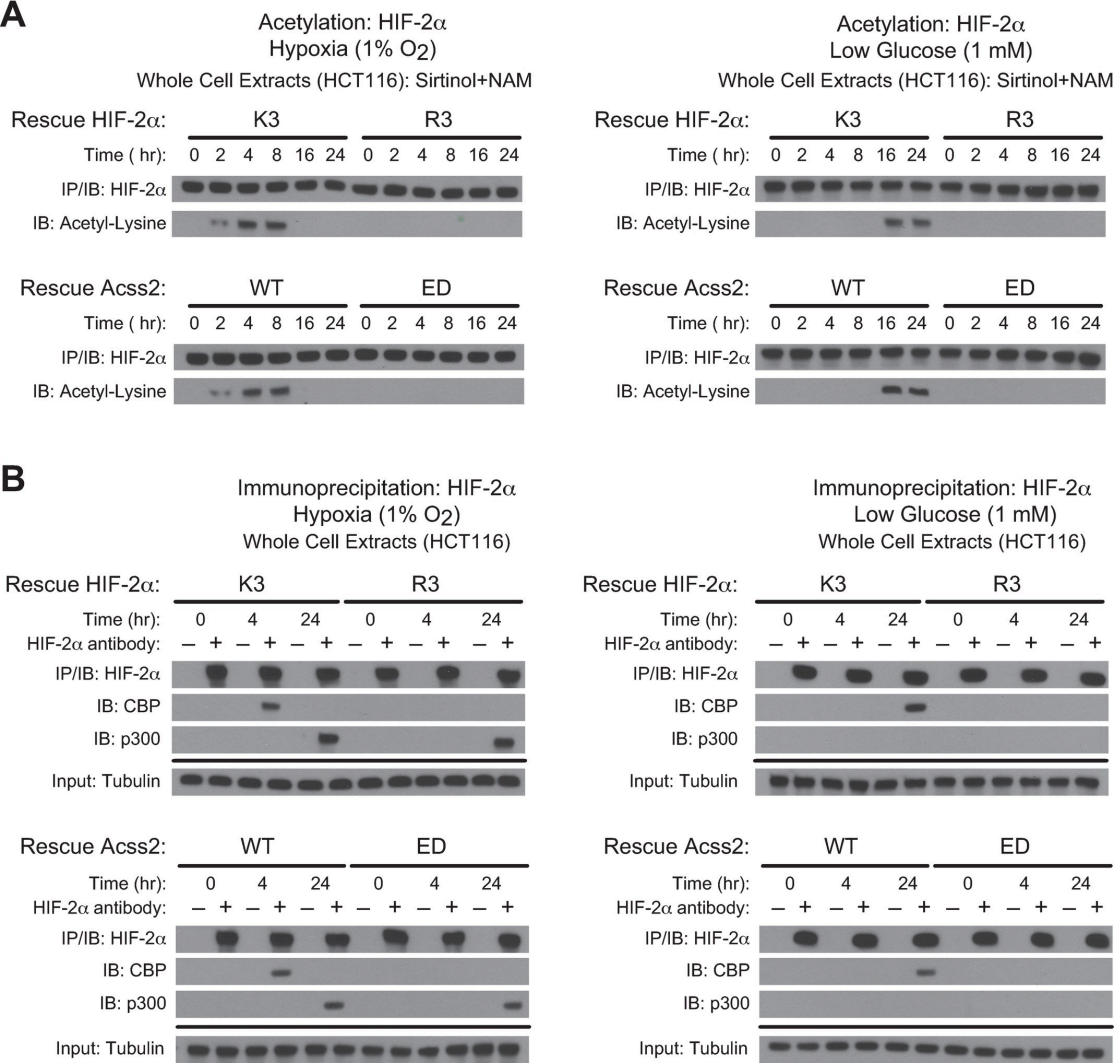
Acss2/Cbp/HIF-2α interactions in HCT116 cells. (A) Acetylation detected by immunoblotting (IB) of ectopic HIF-2α in HIF-2α knockdown HCT116 cells rescued with wild-type (K3) or arginine-lysine substituted mutant (R3) HIF-2α (upper panel), or of endogenous HIF-2α in Acss2 knockdown HCT116 cells rescued with wild-type (WT) or cytosol-restricted mutant (ED) Acss2 (lower panel), following immunoprecipitation (IP) after the indicated period of hypoxia exposure (left side) or glucose deprivation (right side). (B) Interactions of endogenous Cbp or p300 with ectopic HIF-2α in HIF-2α knockdown HCT116 cells rescued with wild-type (K3) or arginine-lysine substituted mutant (R3) HIF-2α (upper panel), or with endogenous HIF-2α in Acss2 knockdown HCT116 cells rescued with wild-type (WT) or cytosol-restricted mutant (ED) Acss2 (lower panel), detected by immunoblotting following immunoprecipitation after hypoxia exposure (left side) or glucose deprivation (right side).

**Fig 4:**
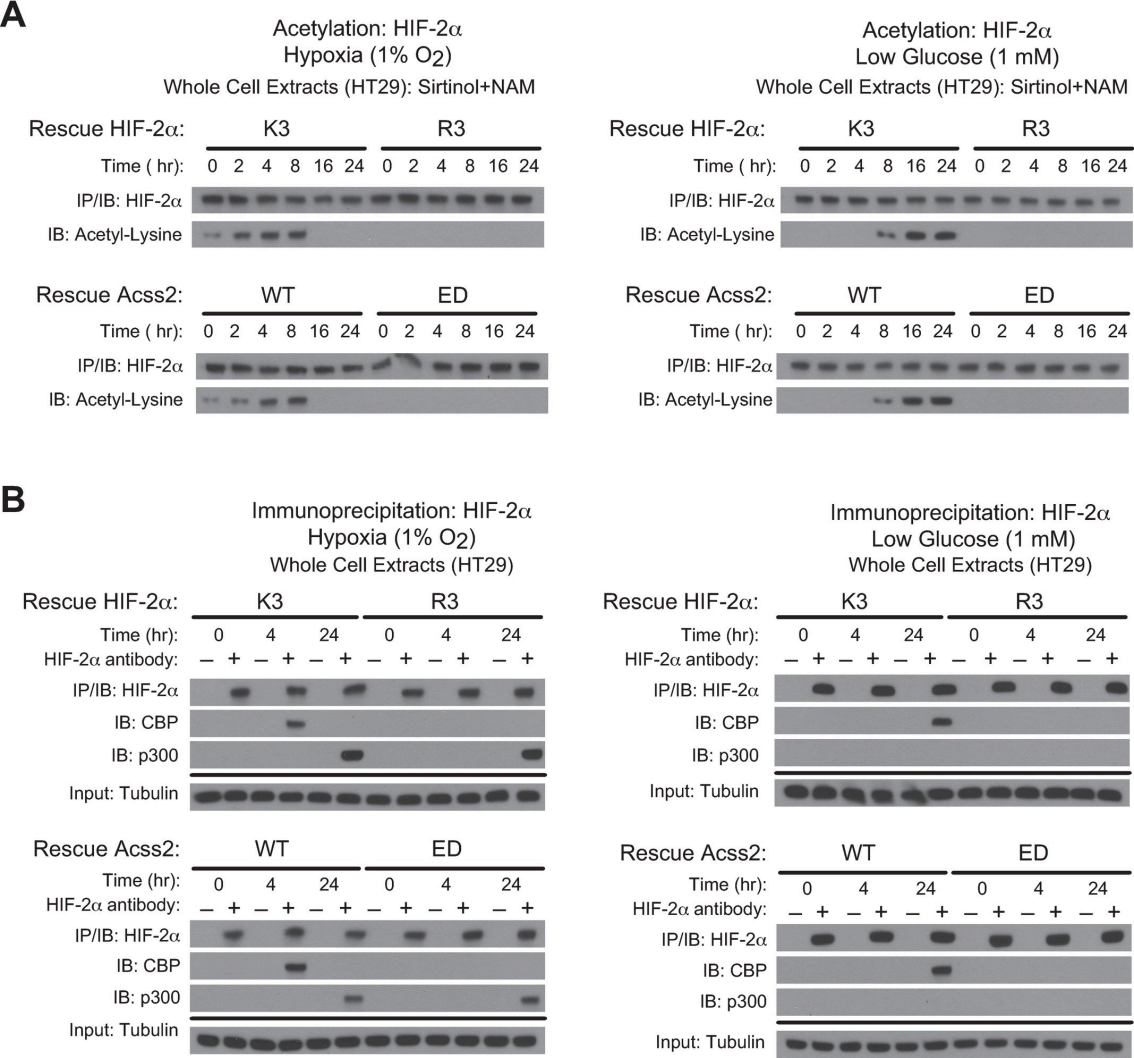
Acss2/Cbp/HIF-2α interactions in HT29 cells. (A) Acetylation detected by immunoblotting (IB) of ectopic HIF-2α in HIF-2α knockdown HT-29 cells rescued with wild-type (K3) or arginine-lysine substituted mutant (R3) HIF-2α (upper panel), or of endogenous HIF-2α in Acss2 knockdown HT29 cells rescued with wild-type (WT) or cytosol-restricted mutant (ED) Acss2 (lower panel), following immunoprecipitation (IP) after the indicated period of hypoxia exposure (left side) or glucose deprivation (right side). (B) Interactions of endogenous Cbp or p300 with ectopic HIF-2α in HIF-2α knockdown HT29 cells rescued with wild-type (K3) or arginine-lysine substituted mutant (R3) HIF-2α (upper panel), or with endogenous HIF-2α in Acss2 knockdown HT29 cells rescued with wild-type (WT) or cytosol-restricted mutant (ED) Acss2 (lower panel), detected by immunoblotting following immunoprecipitation after hypoxia exposure (left side) or glucose deprivation (right side).

Acetylation of HIF-2α is necessary for Cbp/HIF-2α complex formation induced by hypoxia or glucose deprivation. This process requires the presence of specific lysine residues in HIF-2α that are acetylated by Cbp, an intact Cbp acetyltransferase activity, and Acss2 present in the nucleus [7, 8, 15, 16]. We generated stably transformed knockdown/rescue HCT116 and HT29 cells lacking endogenous HIF-2α and expressing shRNA-resistant ectopic wild-type HIF-2α (K3) with intact lysine residues acetylated by Cbp or an arginine substitution HIF-2α mutant (R3) that is not acetylated by and unable to interact with Cbp [7, 8]. We also generated stably transformed knockdown/rescue HCT116 and HT29 cells lacking endogenous Acss2 and expressing ectopic wild-type (WT) mouse Acss2 or a mutant with substitutions (ED) in a putative nuclear localization signal (NLS), which does not translocate to the nucleus following exposure to hypoxia, glucose deprivation, or exogenous acetate. Similar to HT1080 cells, HIF-2α acetylation was not observed in HCT116 and HT29 cells following hypoxia or glucose deprivation when either the R3 HIF-2α mutant or the ED Acss2 mutant were expressed in lieu of their endogenous counterpart (Figs 3A and 4A). Moreover, these mutants also failed to form stable Cbp/HIF-2α complexes during the relevant hypoxia or glucose deprivation time-points (Figs 3B and 4B). However, similar to HT1080 cells, p300/HIF-2α complex formation during late hypoxia (24 hr) was unaffected by these mutations in either HIF-2α or Acss2.

### Acss2/HIF-2 regulates cancer-associated factors in colon cancer cells

**Fig 5:**
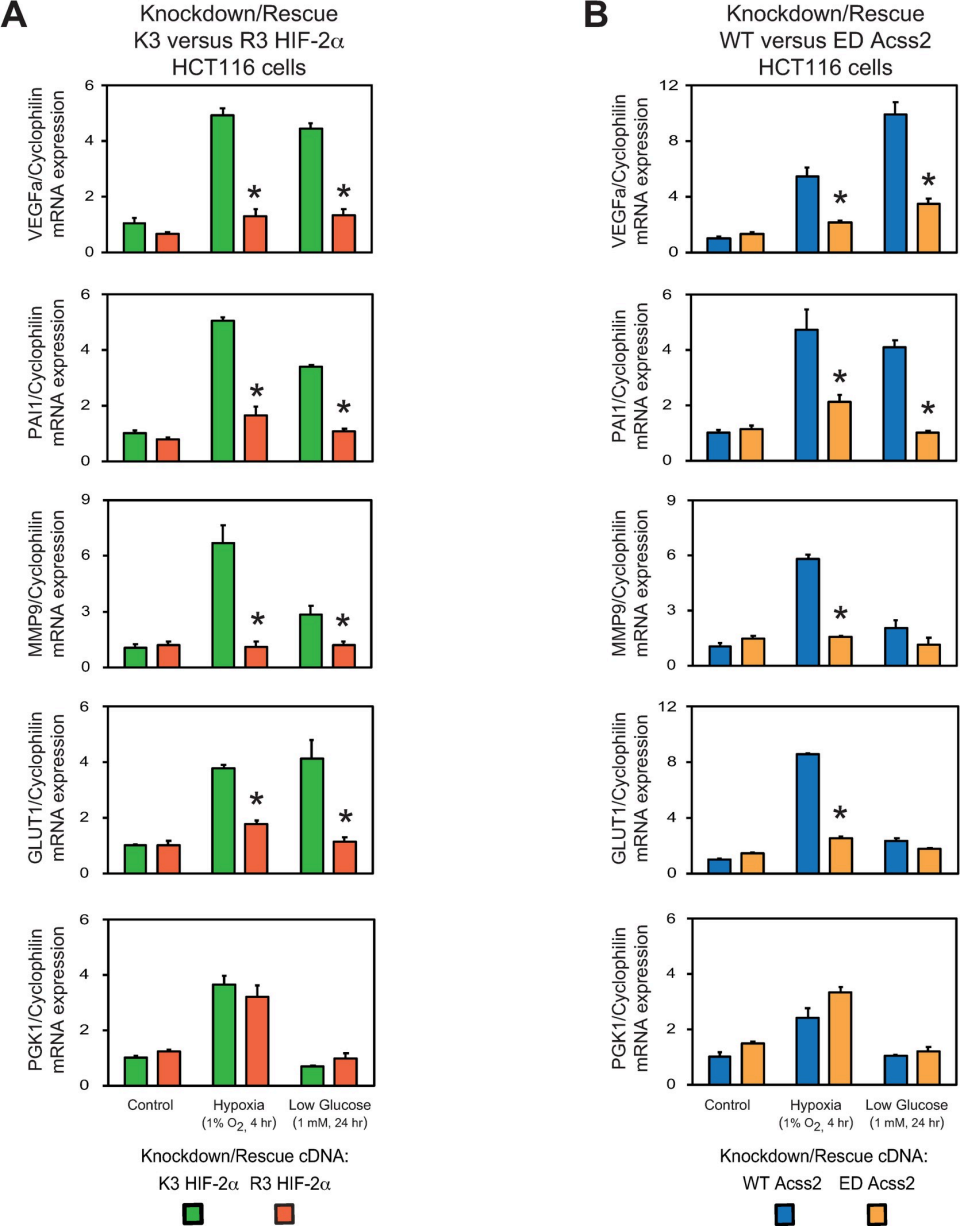
Acss2/HIF-2 regulates cancer-associated factors in HCT116 cells. Semi-quantitative RTPCR measurements of HIF-1 selective (PGK1) target genes in comparison with HIF-2 preferential or HIF-1/HIF-2 co-regulated (VEGFa, PAI1, MMP9, GLUT1) target genes in (A) HIF-2α knockdown HCT116 cells rescued with wild-type (K3) or arginine-lysine substituted mutant (R3) HIF-2α, or (B) Acss2 knockdown HCT116 cells rescued with wild-type (WT) or cytosol-restricted mutant (ED) Acss2, after the indicated period of hypoxia exposure or glucose deprivation. Comparison of samples within a given condition was performed by one-way ANOVA followed by Dunnett’s multiple comparisons test using control shRNA knockdown/control rescue as reference with decreased samples noted (*, P<0.05). Values indicated are means with SD.

**Fig 6:**
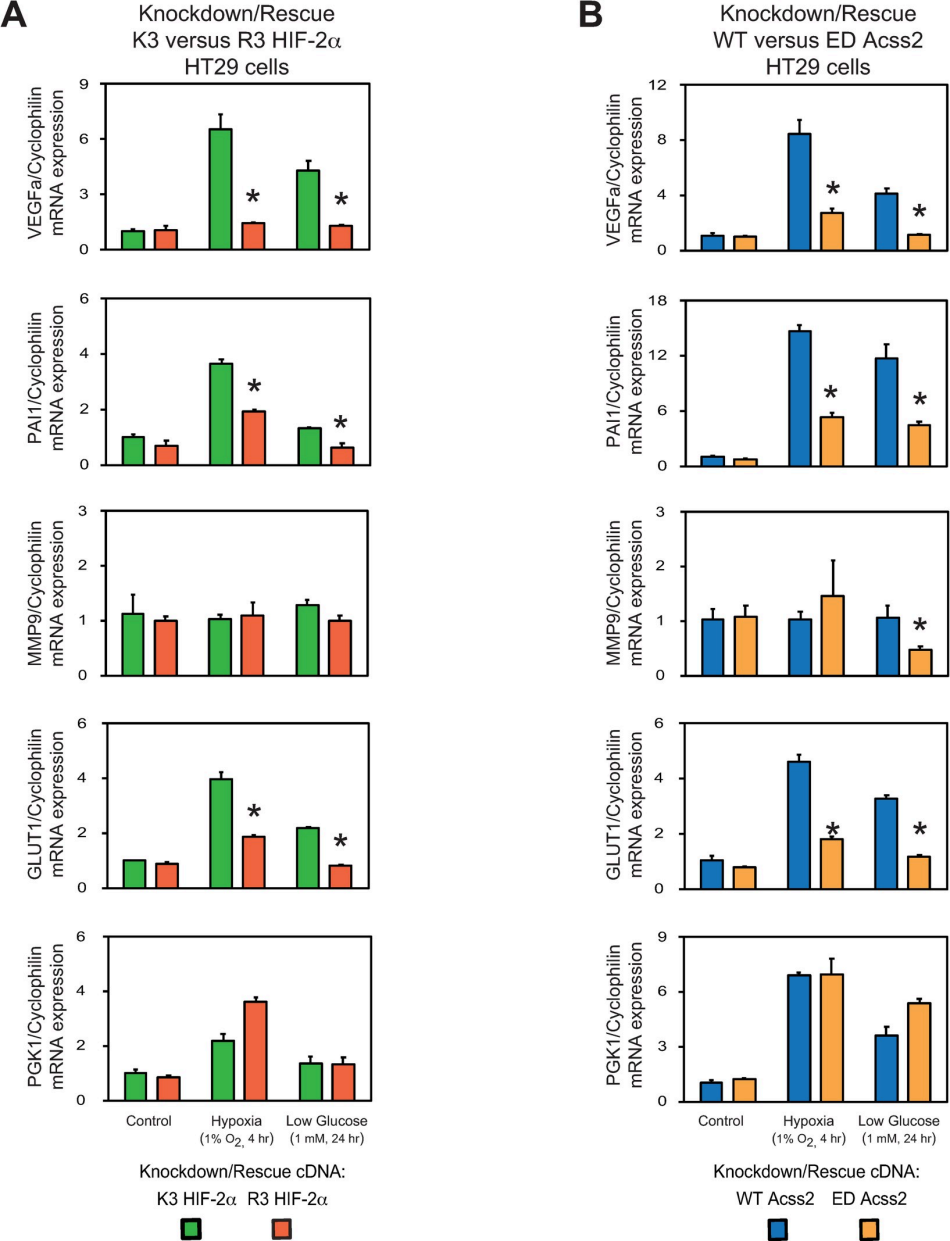
Acss2/HIF-2 regulates cancer-associated factors in HT29 cells. Semi-quantitative RTPCR measurements of HIF-1 selective (PGK1) target genes in comparison with HIF-2 preferential or HIF-1/HIF-2 co-regulated (VEGFa, PAI1, MMP9, GLUT1) target genes in (A) HIF-2α knockdown HT29 cells rescued with wild-type (K3) or arginine-lysine substituted mutant (R3) HIF-2α, or (B) Acss2 knockdown HT29 cells rescued with wild-type (WT) or cytosol-restricted mutant (ED) Acss2, after the indicated period of hypoxia exposure or glucose deprivation. Comparison of samples within a given condition was performed by one-way ANOVA followed by Dunnett’s multiple comparisons test using control shRNA knockdown/control rescue as reference with decreased samples noted (*, P<0.05). Values indicated are means with SD.

We measured HIF target gene induction in stably transformed HCT116 and HT29 knockdown/rescue HIF-2α and Acss2 cells under control or stress conditions (Figs 5 and 6). HIF-2 selective or co-regulated target genes associated with tumor growth and metastasis (*VEGFa*, *PAI1*, *MMP9*, *GLUT1*) were induced in acetylation-intact (K3) HIF-2α and nuclear-localizable (WT) Acss2 knockdown/rescue cells under hypoxia and low glucose conditions. In comparison, HIF-2 target gene induction was blunted in acetylation-defective (R3) HIF-2α or cytosol-restricted (ED) knockdown/rescue cells under these same conditions. In contrast, induction of the HIF-1 selective target gene PGK1 was unaffected or increased in HIF-2α and Acss2 knockdown/rescue cells. The only HIF-2 target gene that exhibited cell line specific characteristics is *MMP9*, which was induced by hypoxia and to a lesser extent under low glucose conditions in K3 HIF-2α and WT Acss2 HCT116 knockdown/rescue cells, but was not induced significantly under these same conditions in K3 HIF-2α and WT Acss2 HT29 knockdown/rescue cells.

### Acss2/HIF-2 regulates colon cancer cell properties

**Fig 7:**
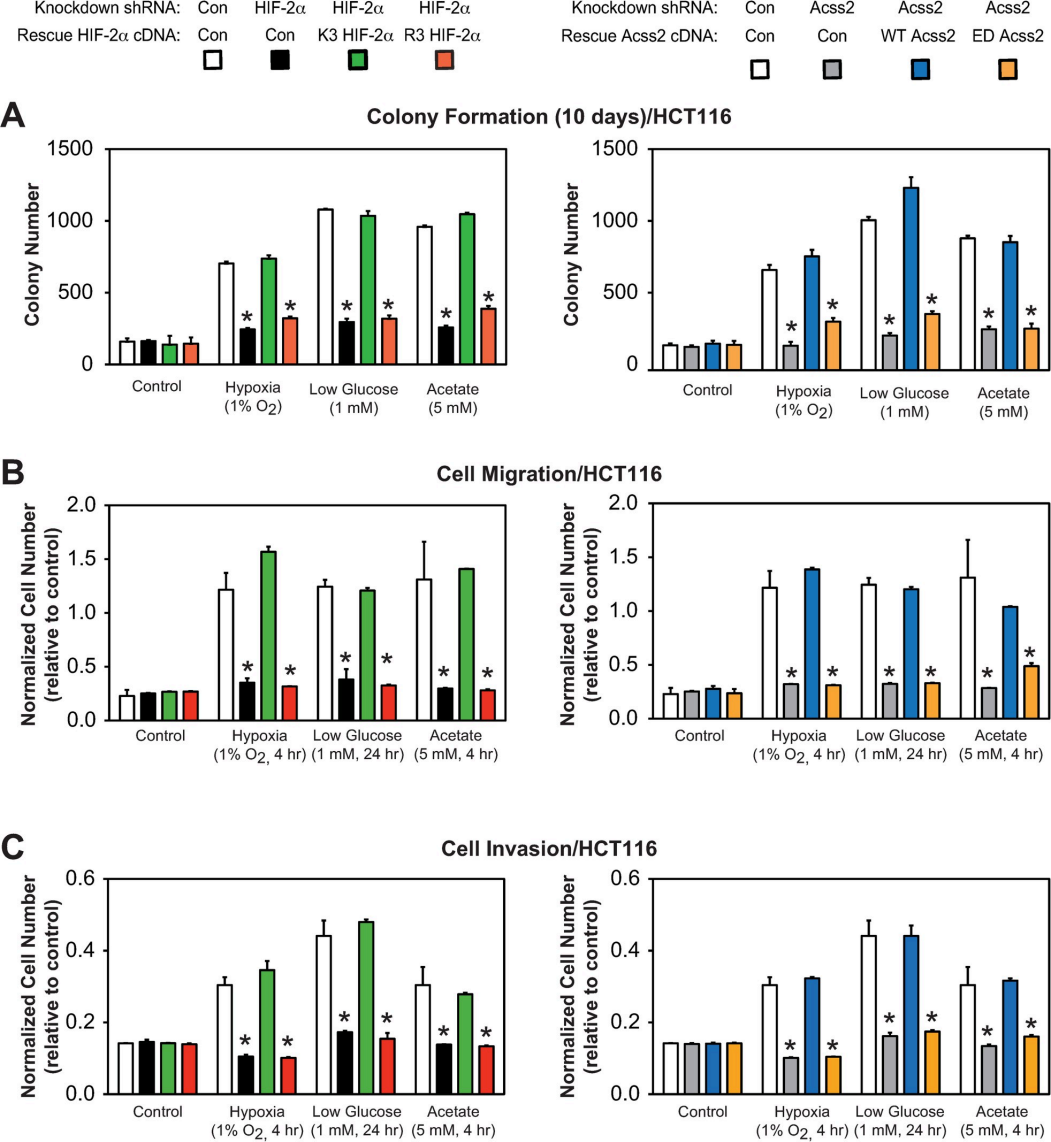
Acss2/Cbp/HIF-2α regulates HCT116 tumor cell properties. (A) Colony formation, (B) Cell migration, or (C) Cell invasion after ten days of stably transformed control or HIF-2α knockdown HCT116 cells rescued with wild-type (K3) or arginine-lysine substituted mutant (R3) HIF-2α (left side), or stably transformed control or Acss2 knockdown HCT116 cells rescued with wild-type (WT) or cytosol-restricted mutant (ED) Acss2 (right side). Cells were exposed to control (21% O_2_, 25 mM glucose), hypoxic (1% O_2_), or low glucose (1 mM) conditions, or were supplemented with acetate (5 mM) (n = 8/treatment/day). Comparison of samples within a given condition on the day of interest for each experiment (colony formation, cell migration, cell invasion) was performed by one-way ANOVA followed by Dunnett’s multiple comparisons test using control shRNA knockdown/control rescue as reference with decreased samples noted (*, P<0.05). Values indicated are means with SD.

**Fig 8:**
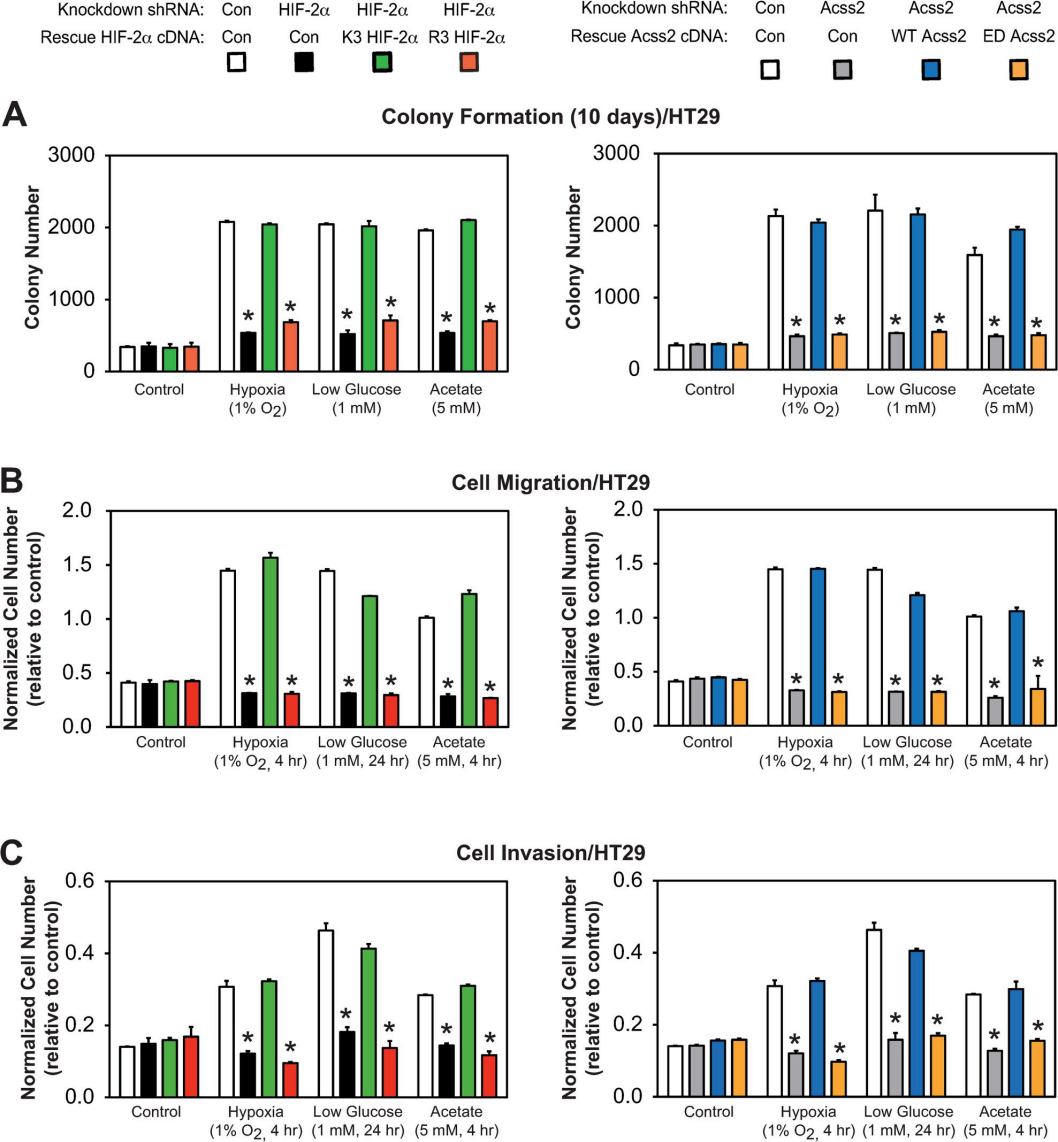
Acss2/Cbp/HIF-2α regulates HT29 tumor cell properties. (A) Colony formation, (B) Cell migration, or (C) Cell invasion after ten days of stably transformed control or HIF-2α knockdown HT29 cells rescued with wild-type (K3) or arginine-lysine substituted mutant (R3) HIF-2α (left side), or stably transformed control or Acss2 knockdown HT29 cells rescued with wild-type (WT) or cytosol-restricted mutant (ED) Acss2 (right side). Cells were exposed to control (21% O_2_, 25 mM glucose), hypoxic (1% O_2_), or low glucose (1 mM) conditions, or were supplemented with acetate (5 mM) (n = 8/treatment/day). Comparison of samples within a given condition on the day of interest for each experiment (colony formation, cell migration, cell invasion) was performed by one-way ANOVA followed by Dunnett’s multiple comparisons test using control shRNA knockdown/control rescue as reference with decreased samples noted (*, P<0.05). Values indicated are means with SD.

We next asked whether *in vitro* tumor cell properties are affected in HCT116 and HT29 knockdown/rescue cells under control, hypoxia, glucose deprivation, or acetate supplemented conditions. We found that colony formation (Figs 7A and 8A), cell migration (Figs 7B and 8B), and cell invasion (Figs 7C and 8C) were all significantly impeded by mutations in either HIF-2α (R3) or Acss2 (ED) that prevent normal Cbp/HIF-2α interactions induced under stress conditions or following acetate supplementation. These mutations in HIF-2α or Acss2 had no or minimal effect on these parameters under control conditions.

### Acetate-augmented colon cancer growth requires Acss2/HIF-2

**Fig 9:**
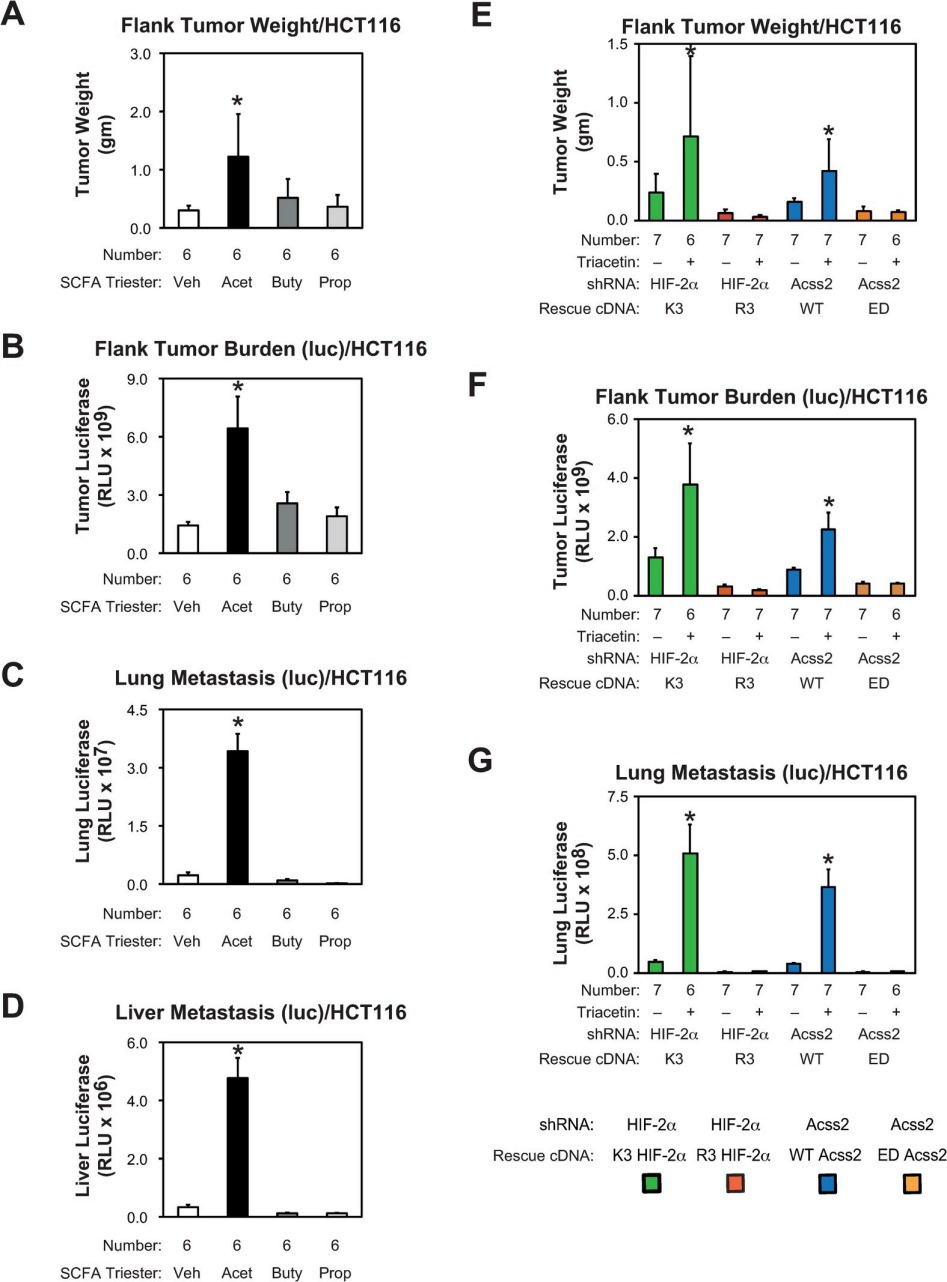
Acetate-augmented HCT116 tumor growth requires Acss2/HIF-2α. (A) Primary tumor weight as well as (B) primary tumor, (C) lung, and (D) liver metastatic burden in nude mice carrying flank tumors derived from luciferase-expressing stably transformed control knockdown HCT116 cells treated by oral gavage with control or acetate (Acet; triacetin), butyrate (Buty, tributyrin), or propionate (Prop; tripropionin) tri-ester (n = 6/treatment). (E) Primary tumor weight as well as (F) primary tumor, and (G) lung metastatic burden in nude mice treated by oral gavage with control or acetate carrying flank tumors derived from luciferase-expressing stably transformed HIF-2α knockdown HCT116 cells rescued with wild-type (K3) or arginine-lysine substituted mutant (R3) HIF-2α, or stably transformed Acss2 knockdown HCT116 cells rescued with wild-type (WT) or cytosol-restricted mutant (ED) Acss2 (n = 6 or 7/treatment). Luciferase activity was assessed by triplicate measurements of tissue extracts. Comparison within each shRNA knockdown/control rescue pair treated with vehicle or acetate (triacetin) was performed by one-tailed unpaired t test with Welch’s correction for groups of equal sample size or by one-tailed Mann-Whitney test for groups of unequal sample size with reductions indicated (*, P<0.05). Values indicated are means with SD (weights) or SEM (luciferase measurements).

**Fig 10:**
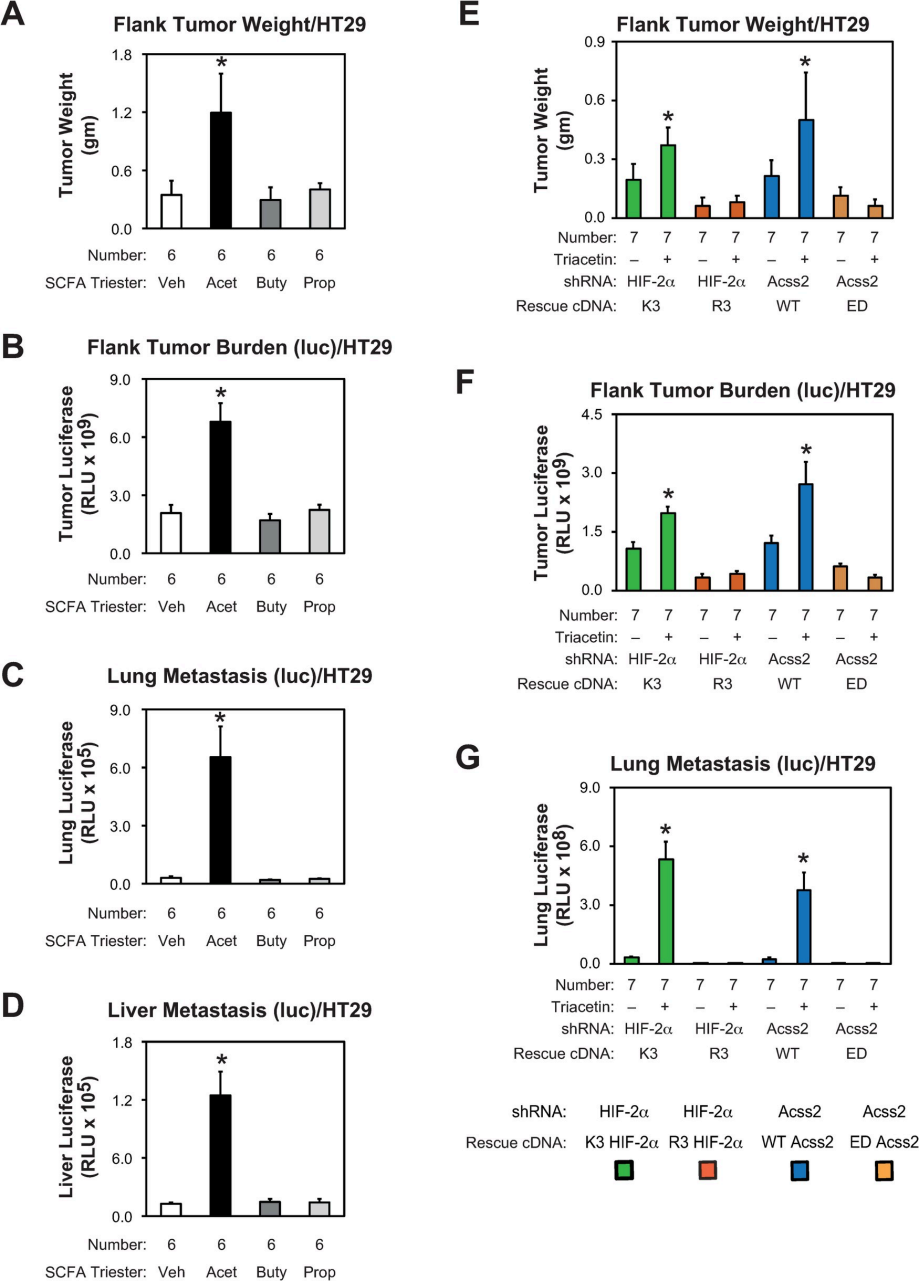
Acetate-augmented HT29 tumor growth requires Acss2/HIF-2α. (A) Primary tumor weight as well as (B) primary tumor, (C) lung, and (D) liver metastatic burden in nude mice carrying flank tumors derived from luciferase-expressing stably transformed control knockdown HT29 cells treated by oral gavage with control or acetate (Acet; triacetin), butyrate (Buty, tributyrin), or propionate (Prop; tripropionin) tri-ester (n = 6/treatment). (E) Primary tumor weight as well as (F) primary tumor, and (G) lung metastatic burden in nude mice treated by oral gavage with control or acetate carrying flank tumors derived from luciferase-expressing stably transformed HIF-2α knockdown HT29 cells rescued with wild-type (K3) or arginine-lysine substituted mutant (R3) HIF-2α, or stably transformed Acss2 knockdown HT29 cells rescued with wild-type (WT) or cytosol-restricted mutant (ED) Acss2 (n = 7/treatment). Luciferase activity was assessed by triplicate measurements of tissue extracts. Comparison within each shRNA knockdown/control rescue pair treated with vehicle or acetate (triacetin) was performed by one-tailed unpaired t test with Welch’s correction for groups of equal sample size or by one-tailed Mann-Whitney test for groups of unequal sample size with reductions indicated (*, P<0.05). Values indicated are means with SD (weights) or SEM (luciferase measurements).

We asked if growth and metastasis of flank tumor derived from parental HCT116 and HT29 cells are affected by oral supplementation with short chain fatty acids. Tumor weight, tumor burden, lung metastasis, and liver metastasis were all increased following supplementation with oral acetate, but not butyrate or propionate, tri-esters (Figs 9A–9D and 10A–10D). The effect on lung and liver metastases was most notable. We next asked if flank tumors derived from stably transformed knockdown/rescue HIF-2α and Acss2 HCT116 and HT29 cells have impaired *in vivo* growth and metastatic potential. We noted metastatic spread to the lung of HCT116 and HT29 flank tumor cells expressing firefly luciferase, which has been reported for mice with HCT116 [20], but not HT29 [21], orthotopic tumors expressing green fluorescent protein. Primary tumor weight, tumor burden, and lung metastases are elevated in mice with flank tumors derived from acetylation-intact (K3) HIF-2α and nuclear-localizable (WT) Acss2 knockdown/rescue cells relative to mice with flank tumors derived from acetylation-defective (R3) HIF-2α or cytosol-restricted (ED) knockdown/rescue cells (Figs 7E–7G and 8E–8G), respectively. Supplementation with the oral acetate compound triacetin augmented growth and metastasis of K3 HIF-2α and WT Acss2 knockdown/rescue flank tumors, but not of R3 HIF-2α or ED Acss2 knockdown/rescue tumors.

### Acss2 is more frequently nuclear localized in human colon cancer

**Fig 11:**
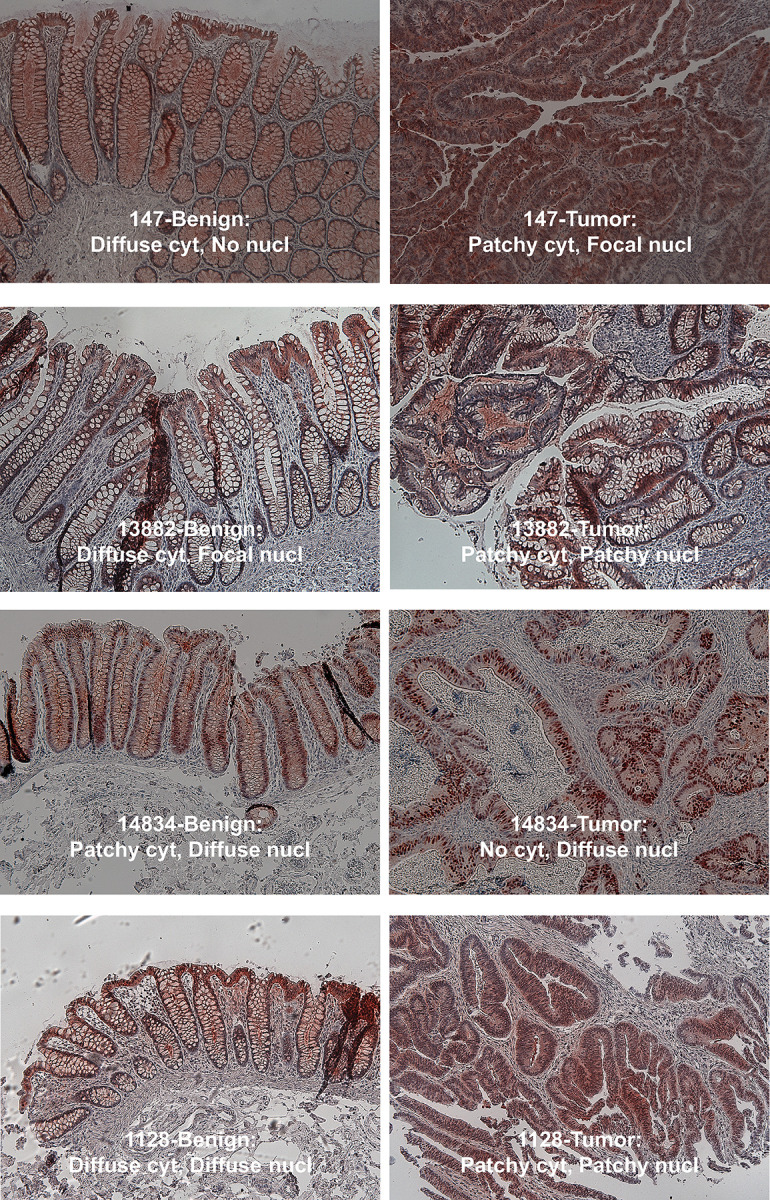
Acss2 is more frequently nuclear localized in human colon cancer. Immunohistochemical staining for Acss2 was performed on sections of paired human colon cancer and normal adjacent tissue samples mounted on the same slide. Low magnification (10x) select images were obtained for representative regions where possible. Scoring of the entire slide as well as select images was performed for intensity and pattern as detailed in methods. Examples of scoring determinations is shown for a subset of select images.

We obtained slides of thirty-four formalin-fixed colon cancers with adjacent normal tissue samples from the University of Texas Southwestern Medical Center Department of Pathology Tissue Resource Core. The de-identified samples retained clinical information associated with staging determined at the time of surgical resection. Tumor and benign tissue for each patient was mounted on a single slide, followed by immunohistochemistry to identify Acss2 protein. The overall intensity as well as pattern of Acss2 immunohistochemical staining in the cytosol and nucleus was first assessed for each slide in a blinded manner using a semi-quantitative scoring system (Fig 11). We next obtained representative images for each slide and scored these images using the same semi-quantitative scoring system as performed for the entire slide. Although details for slide versus select images varied in some cases, largely due to heterogenous staining, the conclusions were similar in both cases. The findings were notable for an decreased cytosolic to nuclear Acss2 ratio in tumor compared to benign samples (Table 1), driven mainly by the absence or reduced presence of Acss2 immunoreactivity in the cytosol of tumor tissue samples.

**Table 1:** Acss2 immunoreactivity is enriched in the nucleus of colon cancer cells.

						Score/Entire Slide						Score/Select Image					
						Benign/Acss2 IHC Intensity			Tumor/Acss2 IHC Intensity			Benign/Acss2 IHC Intensity			Tumor/Acss2 IHC Intensity		
Case	Diagnosis	Stage	TNM Stage	Grade		Cytosolic	Nuclear		Cytosolic	Nuclear		Cytosolic	Nuclear		Cytosolic	Nuclear	
																	
147	invasive adenocarcinoma	T1N0Mx	Stage I	I		1	0		1	1		1	0		1	1	
14834	adenocarcinoma	T2N0M0	Stage I	II		1	3		0	2		1	3		0	2	
936	infiltrating adenocarcinoma	T2N0Mx	Stage I	II		2	2		1	0		2	2		1	1	
12466	adenocarcinoma	T2N0Mx	Stage I	III		1	2		0	2		1	1		1	3	
945	adenocarcinoma	T2N0Mx	Stage I	II		1	2		0	1		1	1		0	1	
783	infiltrating adenocarcinoma with focal mucinous areas	T3N0M0	Stage II	II		2	1		0	0		2	1		0	0	
667	invasive adenocarcinoma	T3N0M0	Stage II	II		1	0		0	1		1	0		0	1	
1128	invasive adenocarcinoma	T3N0M0	Stage II	II		1	1		0	1		1	1		1	1	
811	invasive adenocarcinoma	T3N0M0	Stage II	II		1	0		0	1		1	0		1	1	
13882	adenocarcinoma	T4aN0Mx	Stage II	I		2	1		1	0		2	1		1	2	
850	invasive adenocarcinoma	T4N0M0	Stage II	II		1	1		0	2		1	1		0	2	
685	infiltrating adenocarcinoma	T3N1M0	Stage III	II		1	0		0	0		2	1		0	0	
939	invasive adenocarcinoma	T3N1M0	Stage III	III		1	1		1	0		1	1		1	0	
767	invasive adenocarcinoma	T3N2M0	Stage III	III		1	1		1	0		1	1		1	1	
446	invasive adenocarcinoma	T3N2Mx	Stage III	II		1	1		1	3		1	1		1	3	
662	adenocarcinoma with mucin production	T3N1M1	Stage IV	II		2	0		1	0		2	1		1	1	
917	invasive adenocarcinoma	T3N2M1	Stage IV	II		1	1		1	0		1	0		1	1	
674	invasive adenocarcinoma	T3N2M1	Stage IV	II		1	1		0	1		1	0		0	0	
805	adenocarcinoma with mucin production	T3N2M1	Stage IV	III		1	0		2	1		1	1		2	2	
925	infiltrating adenocarcinoma	T3N2M1	Stage IV	III		1	0		0	0		3	1		1	1	
1521	invasive adenocarcinoma	T4bN2M1	Stage IV	III		2	1		0	3		2	1		0	3	
836	adenocarcinoma	T4N1M1	Stage IV	II		1	1		1	0		1	0		2	2	
840	adenocarcinoma	T4N1M1	Stage IV	II		1	0		0	0		1	0		0	0	
																	
**Intensity: sum**						28	20		11	19		31	19		16	29	
**Ratio Cytosolic/Nuclear intensity**						1.4			0.6			1.6			0.6		

Adenocarcinoma colon cancer samples at various TNM stages and grades as well as benign tissue samples collected at the time of surgery were subjected to immunohistochemistry using antibodies recognizing human Acss2 protein. The intensity of staining in the cytosol and nucleus was assessed by light microscopy for the entire slide as well as for a select 10x representative image using a semi-quantitative scale with staining designated as 0 = none, 1 = faint, 2 = mild, 3 = moderate, 4 = marked, and with the highest number noted as the overall score. A sum of tissue intensity was made for the cytosolic and nuclear scores for tumor and benign tissue samples, and then a ratio of cytosolic/nuclear scores was calculated for benign tissue and tumor samples. A low cytosolic/nuclear ratio indicates a preferential presence of Acss2 in the nucleus.

We also assessed the Acss2 immunohistochemical staining pattern for the entire slide as well as for the select images. For slides, Acss2 immunoreactivity in the cytosol of benign tissues was scored more often as predominantly diffuse whereas Acss2 immunoreactivity in the cytosol of colon cancer samples was more often scored as localized (Table 2). For the select images, the presence of Acss2 in some cases was scored as present whereas it was scored as absent (none) in the corresponding slides. This is likely a reflection of selection and interpretation biases resulting from viewing of a smaller select image versus larger entire slide. However, the pattern of cytosolic Acss2 being predominantly diffuse in benign tissue samples, versus localized and not diffuse when present in colon cancer samples, was evident by the increased diffuse/localized ratio for cytosolic reads of benign tissue for both slide and select image samples.

**Table 2:** Acss2 immunoreactivity exhibits distinct patterns in colon cancer cells.

						Score/Entire Slide						Score/Select Image					
						Benign/Acss2 IHC Pattern			Tumor/Acss2 IHC Pattern			Benign/Acss2 IHC Pattern			Tumor/Acss2 IHC Pattern		
Case	Diagnosis	Stage	TNM Stage	Grade		Cytosolic	Nuclear		Cytosolic	Nuclear		Cytosolic	Nuclear		Cytosolic	Nuclear	
																	
147	invasive adenocarcinoma	T1N0Mx	Stage I	I		D	N		L	L		D	N		L	L	
14834	adenocarcinoma	T2N0M0	Stage I	II		L	D		N	D		L	D		N	D	
936	infiltrating adenocarcinoma	T2N0Mx	Stage I	II		D	L		L	N		D	L		L	L	
12466	adenocarcinoma	T2N0Mx	Stage I	III		D	L		N	D		D	L		L	D	
945	adenocarcinoma	T2N0Mx	Stage I	II		D	L		N	L		D	L		N	L	
783	infiltrating adenocarcinoma with focal mucinous areas	T3N0M0	Stage II	II		D	L		N	N		D	L		N	N	
667	invasive adenocarcinoma	T3N0M0	Stage II	II		D	N		N	L		D	N		N	L	
1128	invasive adenocarcinoma	T3N0M0	Stage II	II		D	L		N	L		D	D		L	L	
811	invasive adenocarcinoma	T3N0M0	Stage II	II		L	N		N	L		L	N		L	L	
13882	adenocarcinoma	T4aN0Mx	Stage II	I		D	L		L	N		D	L		L	L	
850	invasive adenocarcinoma	T4N0M0	Stage II	II		D	L		N	L		D	L		N	L	
685	infiltrating adenocarcinoma	T3N1M0	Stage III	II		D	N		N	N		D	D		N	N	
939	invasive adenocarcinoma	T3N1M0	Stage III	III		D	L		L	N		D	D		L	N	
767	invasive adenocarcinoma	T3N2M0	Stage III	III		L	L		L	N		D	D		L	L	
446	invasive adenocarcinoma	T3N2Mx	Stage III	II		L	L		L	L		L	L		L	L	
662	adenocarcinoma with mucin production	T3N1M1	Stage IV	II		D	N		L	N		D	D		L	L	
917	invasive adenocarcinoma	T3N2M1	Stage IV	II		D	L		L	N		D	L		L	L	
674	invasive adenocarcinoma	T3N2M1	Stage IV	II		L	L		N	N		L	N		N	N	
805	adenocarcinoma with mucin production	T3N2M1	Stage IV	III		L	N		L	L		L	L		L	L	
925	infiltrating adenocarcinoma	T3N2M1	Stage IV	III		D	N		N	N		D	D		L	L	
1521	invasive adenocarcinoma	T4bN2M1	Stage IV	III		D	D		N	L		D	D		N	L	
836	adenocarcinoma	T4N1M1	Stage IV	II		D	L		L	N		D	N		D	D	
840	adenocarcinoma	T4N1M1	Stage IV	II		L	N		N	N		D	N		N	N	
																	
**Number of None**						0	8		13	12		0	6		9	5	
**Number of Diffuse**						16	2		0	2		18	8		1	3	
**Number of Localized**						7	13		10	9		5	9		13	15	
**Ratio of Number Diffuse/Number Localized**					** **	2.3	0.2		0.0	0.2		3.6	0.9		0.1	0.2	** **

The pattern of Acss2 immunoreactivity for the adenocarcinoma colon cancer and benign tissue samples was gauged by light microscopy for the entire slide as well as for a select 10x representative image using a qualitative scale as follows: N = none, D = diffuse, L = localized. A sum of pattern scores was made for the diffuse and localized pattern in both the cytosolic and nuclear compartments for benign tissue and tumor samples. A ratio of diffuse/localized scores was then calculated for each subcellular compartment. A high diffuse/localized ratio indicates a preferential diffuse Acss2 expression pattern.

## Discussion

Cytosolic and nuclear acetyl CoA pools are considered sequestered in eukaryotic cells [22, 23]. Communication between these pools provides an opportunity to link changes in cytosolic metabolism with nuclear signal transduction events [24]. The translocation or enrichment of cytosolic acetyl CoA generators into the nucleus establishes a molecular mechanism to establish such a dynamic relationship. We have shown that increased acetate levels following hypoxia or low glucose exposure is coincident with increased nuclear Acss2 levels [15, 16]. Furthermore, these events coincide or precede Cbp-mediated HIF-2α acetylation and Cbp/HIF-2α complex formation. Exogenous acetate supplementation also results in nuclear localization of Acss2 and subsequent activation of these processes. However, forced nuclear expression of Acss2 is not sufficient to augment HIF-2 signaling. Thus, increased intracellular acetate triggers Acss2 translocation into or enrichment in the nucleus, but is also required to generate acetyl CoA for nuclear acetylation reactions mediated by Cbp and possibly other acetyltransferases.

In eukaryotes, acetate and hypoxia signaling are linked through the Acss2/Cbp/Sirt1/HIF-2 signaling axis. Acss2 regulates dynamic HIF-2α acetylation, Cbp/HIF-2α complex formation, Cbp/HIF-2 signaling, and tumor cell growth and metastasis [14, 15]. Knockdown and rescue using a cytosol-restricted Acss2 mutant protein (ED) does not restore these activities whereas rescue with a wild-type Acss2 protein does [14]. This same pattern is found following knockdown and rescue using an acetylation-defective HIF-2 mutant (R3) that does not form stable complexes with Cbp [14]. Our current model is that Acss2-induced recruitment of Cbp to HIF-2α results in acetylation of HIF-2α as well as of local histones in proximity to HIF-2 target genes. Whether Cbp interacts with other transcriptional regulators in an Acss2-selective and similar manner as seen with HIF-2α remains to be determined.

The similar and favorable outcomes for mice with HCT116 and HT29 derived flank tumors marked by impaired Acss2/HIF-2 signaling suggest that targeting this pathway may have broad efficacy in colon cancer. Moreover, the similar response of HCT116 and HT29 also has important implications for colon cancers marked by different driver mutations. HCT116 and HT29 differ in growth characteristics [25, 26]. HCT116 is considered a more aggressive and non-differentiating colorectal cancer cell line whereas HT29 exhibits limited differentiation potential [27]. The K-Ras proto-oncogene is frequently mutated in colorectal cancer [28, 29]. HCT116 possesses a mutant Ras protein and a hyperactive Ras pathway as a result, whereas HT29 possesses wild-type Ras and exhibits very low Ras activity. Possibly related to this observation, the prevalence of cancer stem cells in the HCT116 cell population is greater compared to the HT29 cell population [27].

With respect to its effect on HIF signaling, activated K-Ras induces a cellular response that may involve or impact both HIF-1 and HIF-2. Knockdown of mutant K-Ras in HCT116 cells impairs hypoxic induction of HIF-1α [30], suggesting that activated K-Ras may augment HIF-1 signaling through effects on HIF-1α protein stability. Restoration of wild-type K-Ras in HCT116 cells results in a reduction in both HIF-1 and HIF-2 target gene expression, which implies that HIF-2 signaling may also be active in HCT116 cells [31]. Activating mutations in the B-Raf oncogene also are prevalent in colon cancer. However, activating mutations in K-Ras and B-Raf are mutually exclusive in colon cancer [32–34]. With respect to HIF signaling, HT29 cells possess mutant B-Raf and knockdown of mutant B-Raf results in reduced protein levels of HIF-2α [30]. Thus, major drivers of colon cancer activate HIF signaling, possibly mechanistically in a more direct HIF isoform-specific manner. Activation of either HIF-1 or HIF-2 is likely to have tumor enhancing effects regardless of the nature of the activated proto-oncogene, which is in part due to crosstalk between these two HIF isoforms at many HIF target genes.

Acss2 has also been implicated in colon cancer growth, although the studies are more limited compared with HIF and colon cancer. Knockdown of Acss2 in several colon cancer cell lines enhanced cell death under hypoxia and slowed tumor growth in mice [35], which was attributed to decreased Acss2-dependent cytosolic acetate uptake and lipid incorporation in tumor cells [35, 36]. The role of acetate in colon cancer biology is less obvious. A recent study focusing on metabolic features of HCT116 and HT29 under normoxic, hypoxic, or acetate-supplemented conditions found that acetate induces growth arrest through effects on mitochondrial function [37], which is supporting evidence for earlier *in vivo* studies indicating that enhanced delivery of acetate reduces tumor size [38]. Our current and previous studies indicate that Acss2 facilitates cancer growth in part via effects on HIF-2 signaling and these Acss2-dependent effects are augmented with supplemental acetate admistration [14, 15]. Moreover, the effect of impeding Acss2/HIF-2 signaling on growth and metastasis of HCT116 and HT29 derived colon cancer flank tumors in the current study is impressive.

The immunohistochemical analyses of Acss2 in human colon cancer samples and their benign neighboring tissue is noteworthy in several respects. Acss2 immunoreactivity was detected in most human colon cancer samples that we examined and was preferentially nuclear. The effect of the solid tumor microenvironment, combined with its anatomical location that results in exposure to high acetate levels generated by the gut microbiome, may explain why Acss2 protein localization in colon cancer samples is preferentially nuclear [39]. Solid tumor cells experience hypoxia and glucose deprivation, which result in elevated acetate levels that promote Acss2 expression in the nucleus [14–16]. However, some human colon cancer samples in our study had no or minimal Acss2 immunoreactivity in either the cytosol or nucleus. This may be due to differences in cellular architecture that obscure Acss2 immunoreactivity, such as aneuploidy, or to use of an alternative acetyl CoA generator, ATP citrate lyase, for cytosolic and possibly nuclear acetyl CoA generation. Alternatively, Acss2 protein may be absent due to effects on protein stability.

Another finding from the Acss2 immunohistochemical analyses was the predominantly diffuse pattern of Acss2 in the cytosol of benign tissue versus a more localized pattern in the cytosol of colon cancer samples. Acss2 staining was heterogenous in some samples. Although some heterogenous staining may be artifactual, it is possible that Acss2 steady-state protein levels vary in different anatomical portions of the colon cancer solid tumor. A mutant Acss2 protein (CYT), which harbors a substitution mutation (ED) in a putative nuclear localization signal (NLS), is cytosolic-restricted and enzymatically active when over-expressed in HT1080 cells [14]. However, this same and other mutant Acss2 proteins are extremely unstable when expressed endogenously in mice [40, 41] and exhibit lower levels even when over-expressed in cancer cell lines. The instability of these mutant Acss2 proteins is due to unmasking of potent protein destabilization elements (PDE), which are normally caged in wild-type Acss2 protein [40]. Whether PDE remain masked in wildtype Acss2 expressed in solid tumors, or whether they are unmasked in some regions of solid tumors due to local effects of the tumor microenvironment, remains to be determined.

Colon cancer tumors are exposed to the highest levels of exogenous acetate in the body due to their proximity to the gastrointestinal microbiome. Acss2 and Acss2/HIF-2 signaling may have a particularly prominent role in growth and metastasis of cancers where extracellular acetate levels are elevated or where acetate is a primary substrate for growth [42, 43]. Indeed, acetate supplementation promotes *in vitro* migration and invasion of HCT116 and HT29 colon cancer cells, which is blunted with inhibition of Acss2 or HIF-2 signaling. Oral acetate supplementation also augments *in vivo* HCT116 and HT29 flank tumor growth and metastases, which likewise decreases significantly when Acss2 or HIF-2 signaling is impaired. We found that basal as well as acetate-augmented growth and metastasis of HCT116 and HT29 flank tumors in mice depends upon Acss2/HIF-2 signaling. Based on these data, inhibition of Acss2 or HIF-2 as a monotherapy may be advantageous in colon cancer therapies. Moreover, because of their positions in the same signaling axis, targeting both Acss2 and HIF-2 may be a particularly attractive treatment strategy for colon cancer patients with more advanced disease.

## Supporting information

S1 Fig Original scans of immunoblots. Scans of all films for immunoblots presented in this study.
(PDF)

S2 Fig Select images from colon cancer and benign tissue samples. Light microscopy of select images (10x) from Acss2 immunohistochemical-stained colon cancer and benign adjacent tissue samples used to generate data select image scoring in Tables 1 and 2. Whenever possible, images were obtained to document representative fields. In cases of slides with overall minimal Acss2 immunoreactivity, fields were chosen where some Acss2 immunohistochemical staining was evident.
(SVGZ)

S1 Table Annotated raw data for chart data. Raw data used for all charts presented in this study.
(XLSX)
