# Supplementary material for: Acss2/HIF-2 signaling facilitates colon cancer growth and metastasis
Source: PLoS One. 2023 Mar 2;18(3):e0282223. doi: 10.1371/journal.pone.0282223 (PMC9980813; doi:10.1371/journal.pone.0282223)

HIF1a and HIF2a expression in HCT116 and HT29 cells

Hypoxia (HYP), Low Glucose (LG)

IB: HIF-1α

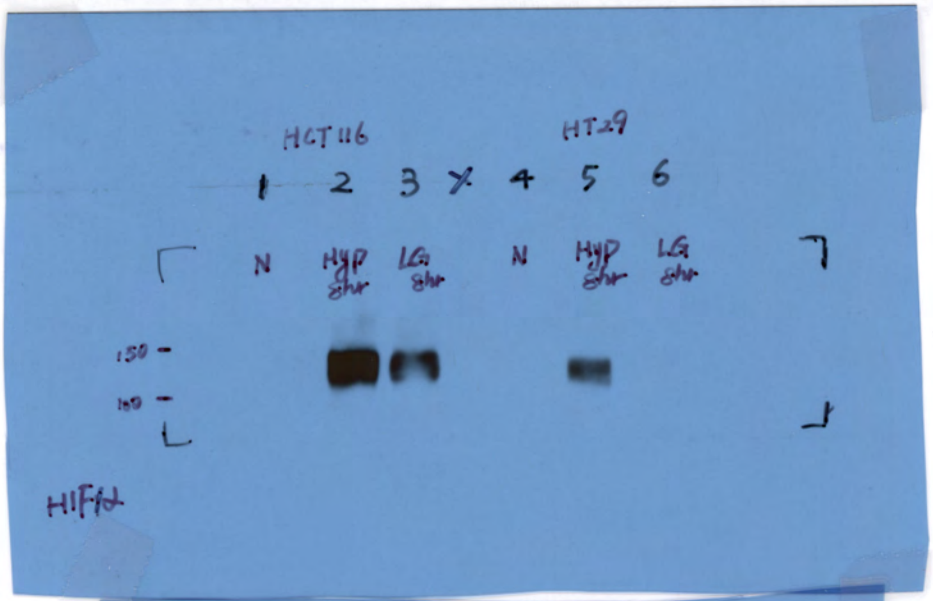


Fig 1A, 2A

IB: HIF-2α (SHORT EXP)

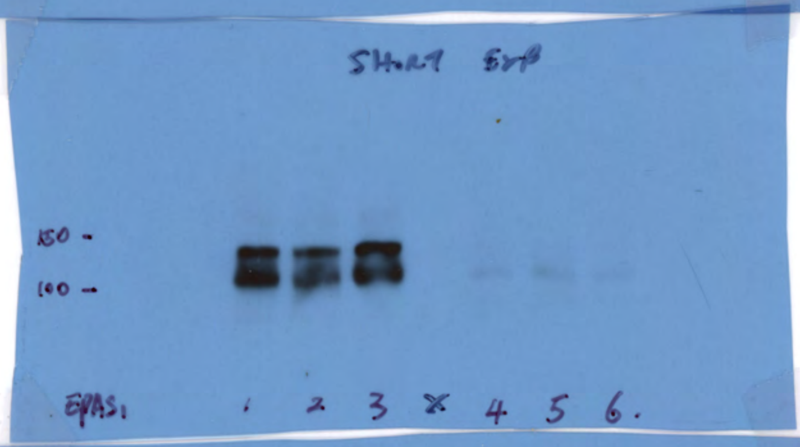


IB: HIF2α (long Exp)

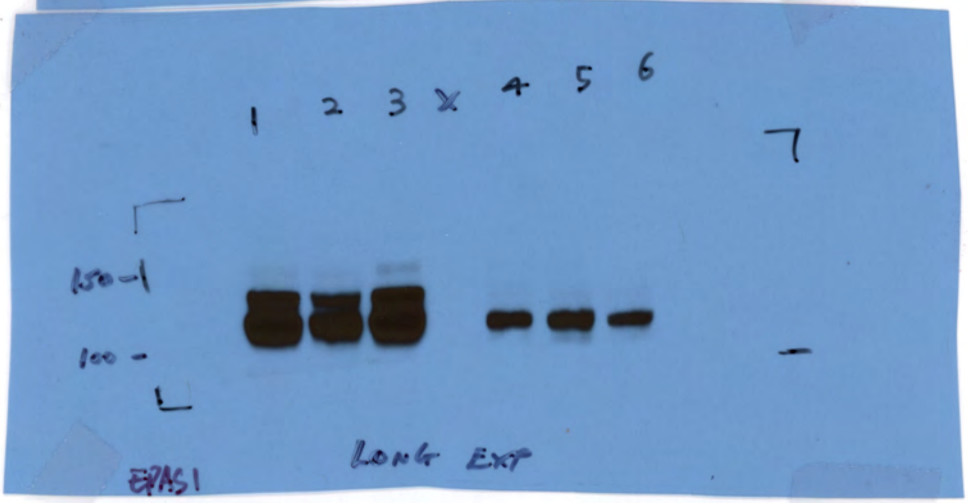


Fig 1A, 2A

IB: α-TUBULIN (MEMBRANE CUT FROM HIF-2α IB)

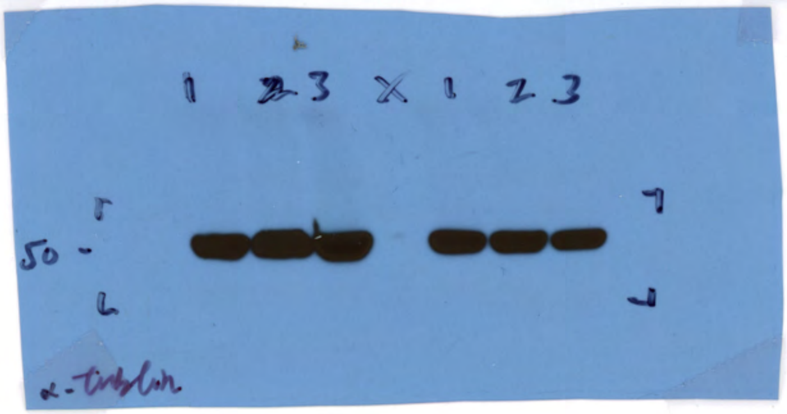


Fig. 1A, 2A

Acetylation TC_ Hyp, LG_ endo HIF2a_ IP: sp_ IB: endo HIF2a, Ack / HCT116 cells

Hypoxia (Hyp)

IP: HIF-2α
IB: ACETYL-LYSINE

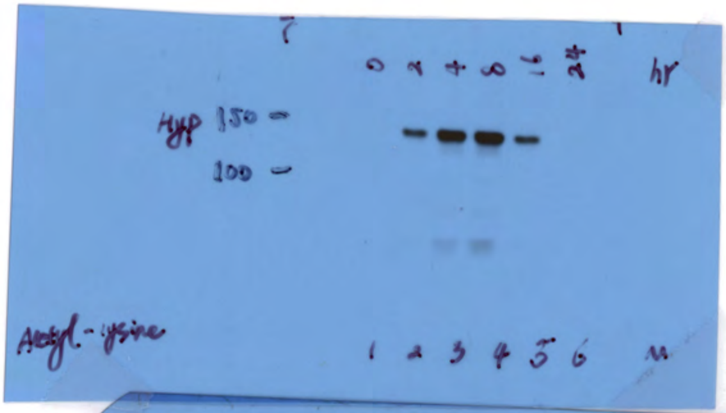


Fig 1C (left panel)

IP = HIF-2α
IB = HIF-2α

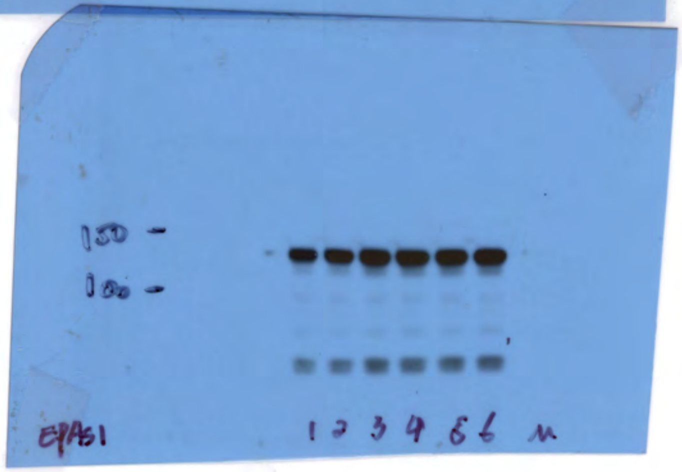


Low Glucose (LG)

IP: HIF-2α
IB: ACETYL-LYSINE

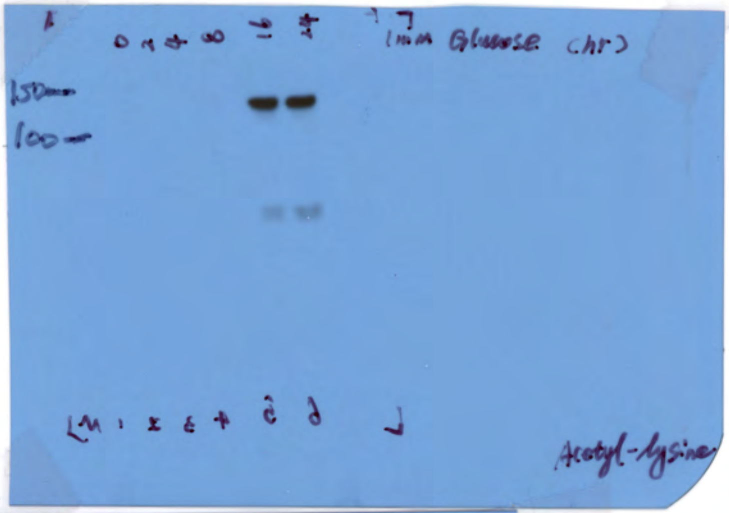


Fig 1C (right panel)

IP: HIF-2α
IB: HIF-2α

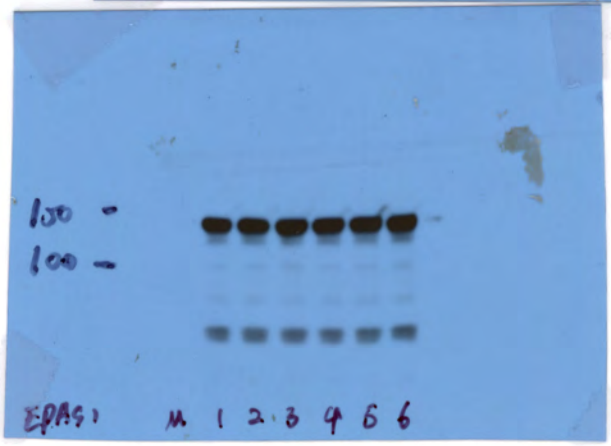

CoIP (3 time-points)_
Normoxia, Hypoxia _ endo
HIF 2α, cbp, p300_IP:
HIF2α _ IB: HIF2α,
Cbp, p300
HCT116 WHOLE CELL EXTRACTS

Fig. 1D
(left panel)

Hypoxia
(HYP)

IP: HIF-2α
IB: CBP

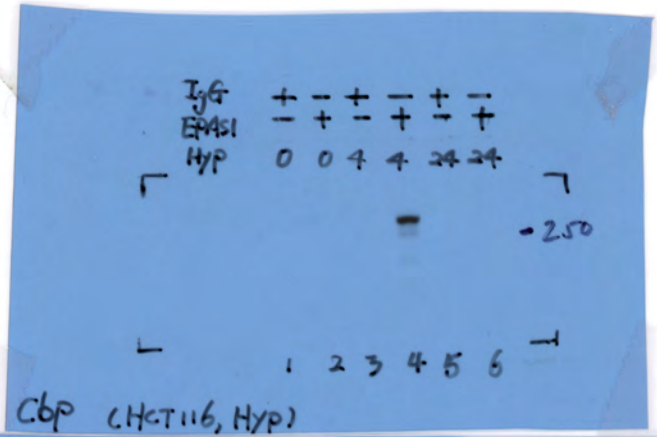


IP: HIF-2α
IB: p300

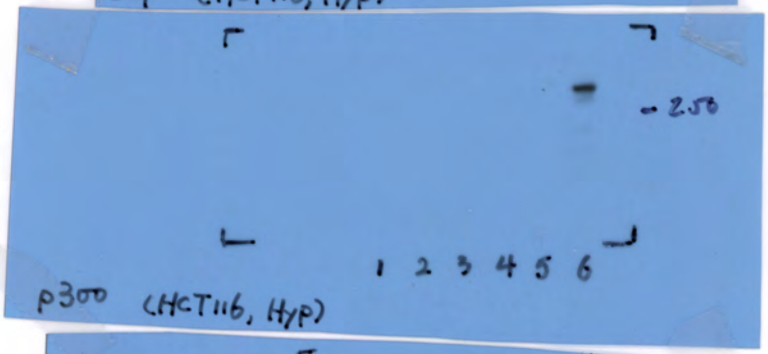


IP: HIF-2α
IB: HIF-2α

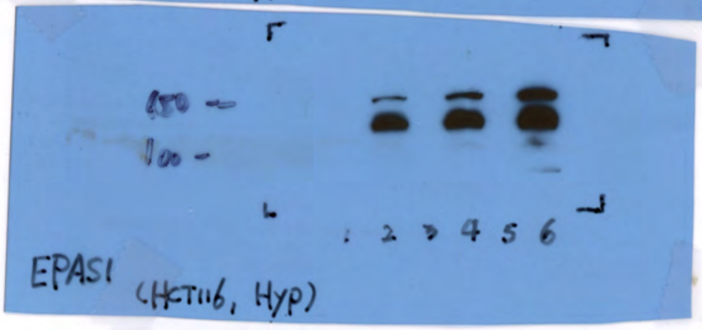


LOAD
IB: CBP

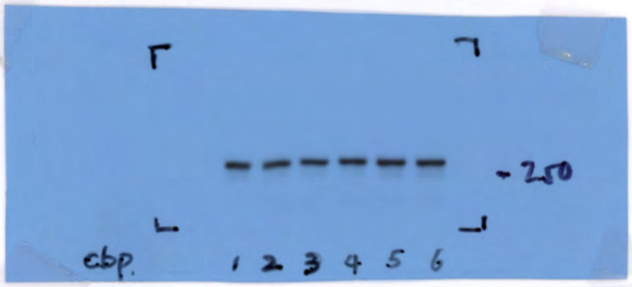


LOAD
IB: p300

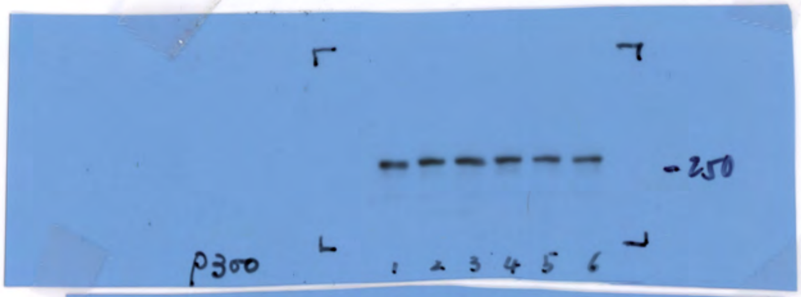


LOAD
IB: α-TUBULIN

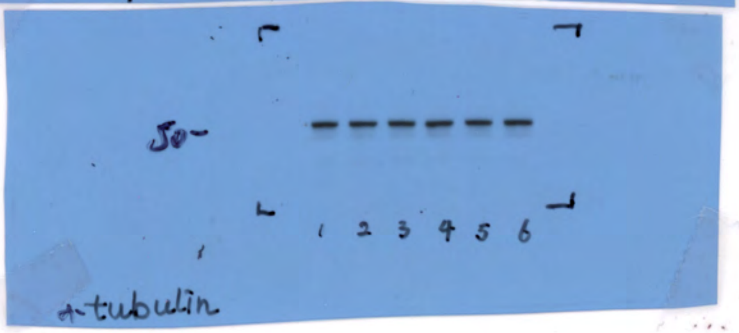

CoIP (3 time-points)_
Norm, LG- endo HIF2α, cbp, p300_ IP: HIF2α-
IB: HIF2α, cbp, p300
HCT116 WHOLE CELL EXTRACTS

Low Glucose (LG)

Fig. 1D (right panel)

IP: HIF-2α
IB: CBP

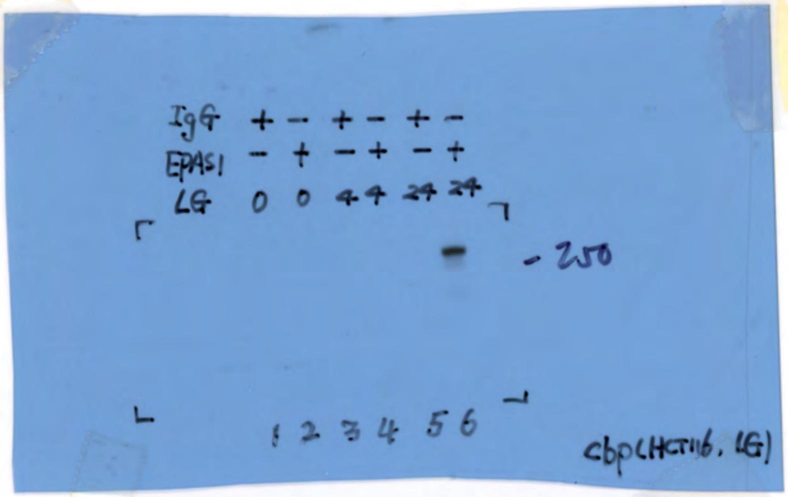


IP: HIF-2α
IB: p300

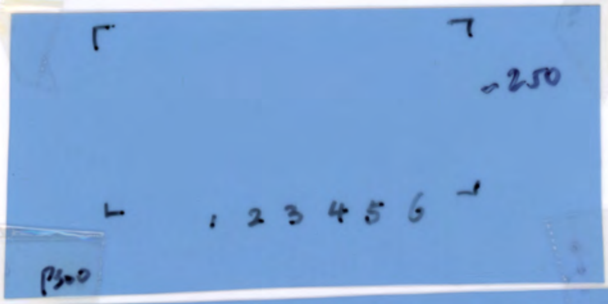


IP: HIF-2α
IB: HIF-2α

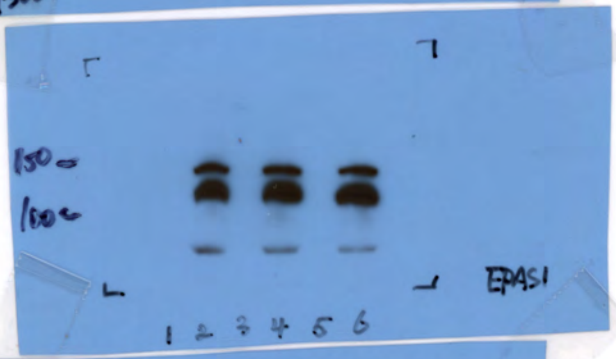


LOAD
IB: CBP

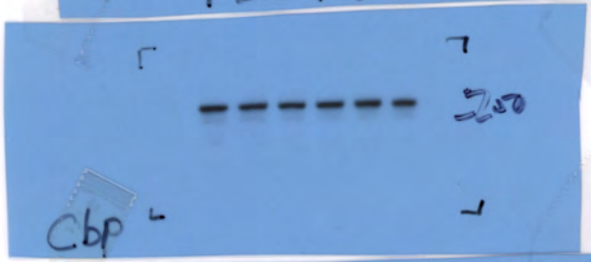


LOAD
IB: p300

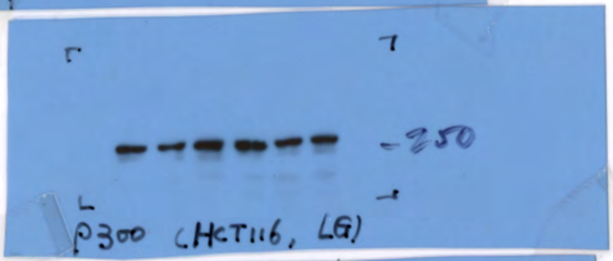


LOAD
IB: α TUBULIN

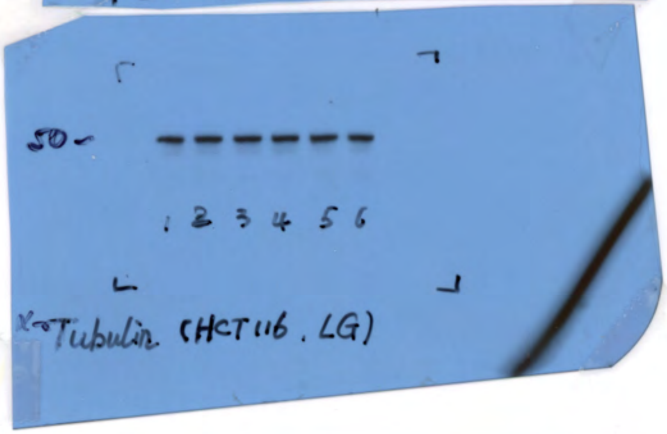

Acetylation TC – Hyp, LG – endo H2F2α – IP: SP – IB: endo H2F2α, Ack / HT29 cells

Hypoxia (HYP)
IP: HIF-2α
IB: ACETYL-LYSINE

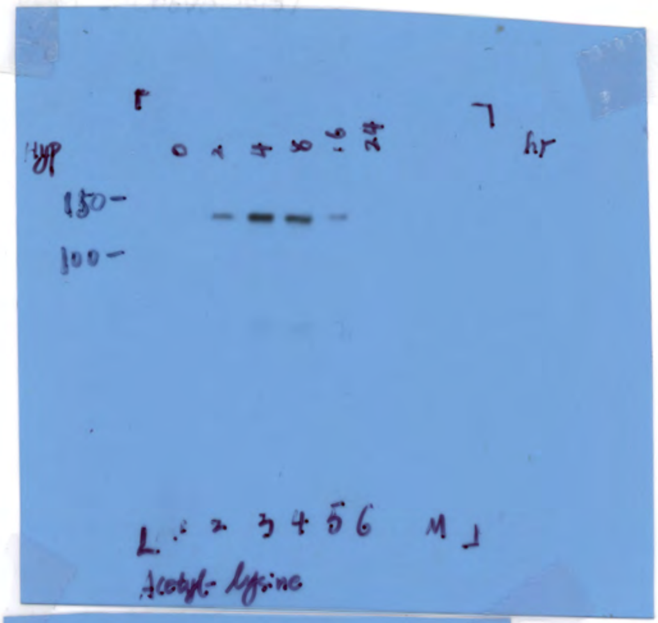


Fig 2C (left panel)

IP: HIF-2α
IB: HIF-2α

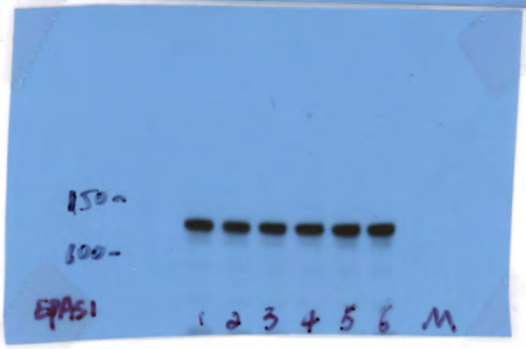


Low Glucose (LG)
IP: HIF-2α
IB: ACETYL-LYSINE

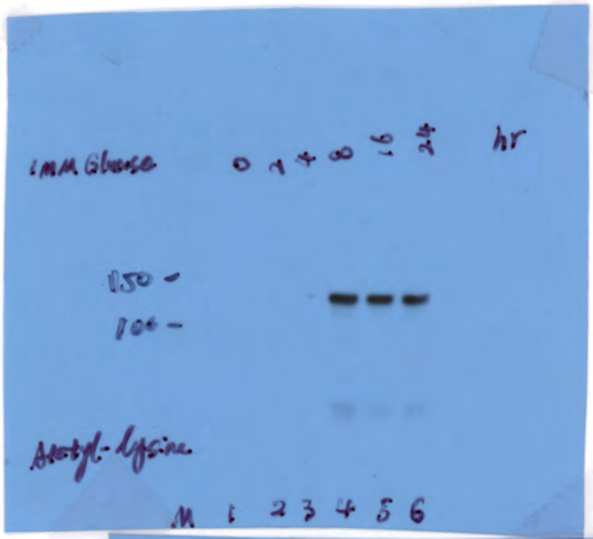


Fig 2C (right panel)

IP: HIF-2α
IB: HIF-2α

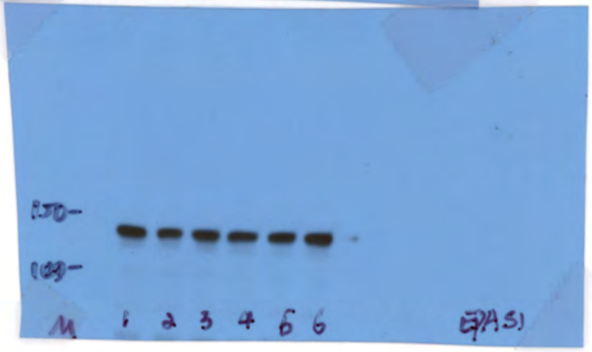

Fig. 2D (left panel)

CoIP (3 time-points)
- Normoxia, Hypoxia
- endo HIF2α, cbp, p300 - IP: HIF2α - IB: HIF2α, cbp, p300 in HT29 WHOLE CELL EXTRACTS

Hypoxia (Hyp) IP: HIF-2α IB: CBP

IP: HIF-2α IB: p300

IP: HIF-2α IB: HIF-2α

LOAD IB: CBP

LOAD IB: p300

LOAD IB: α-TUBULIN

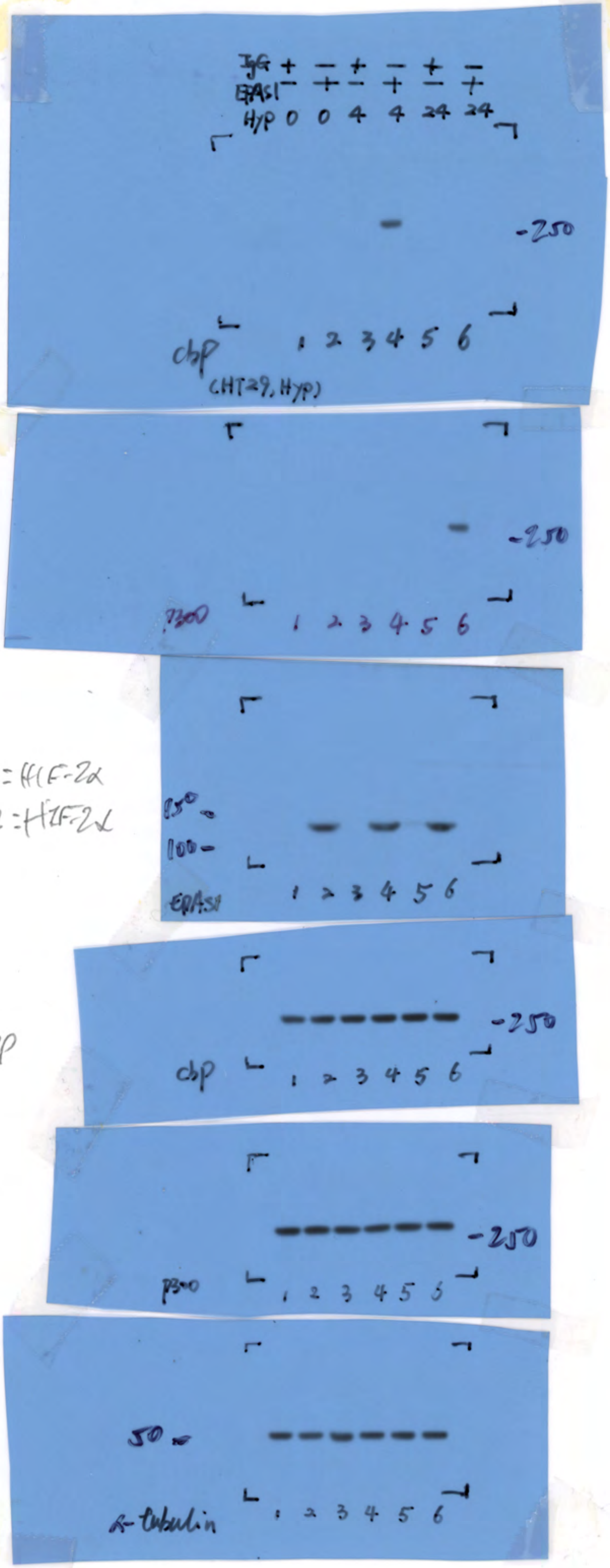

CoIP (3 time-points)_
Normoxia, Lowglucose_
endo HIF2α, cbp, p300_
IP: HIF2α _ IB: HIF2α,
cbp, p300 in HT-29 cells
HT29 WHOLE CELL EXTRACTS

Fig. 2D
(right panel)

Low Glucose (LG)
IP: HIF-2α
IB: CBP

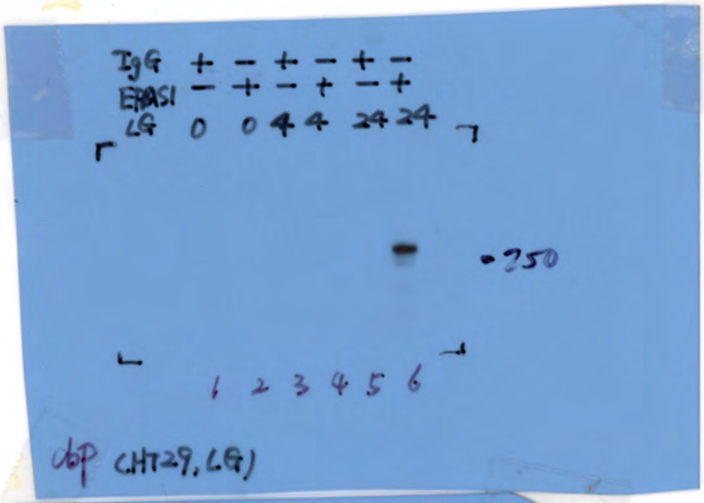


IP: HIF-2α
IB: p300

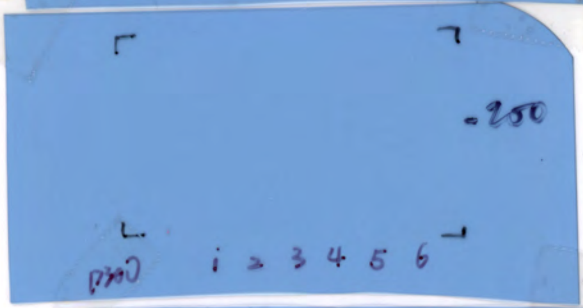


IP: HIF-2α
IB: HIF-2α

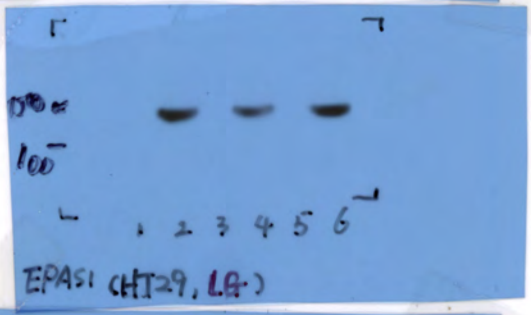


LOAD
IB: CBP

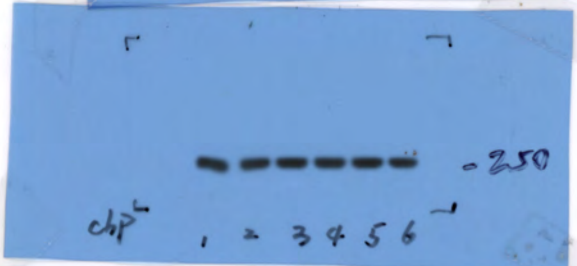


LOAD
IB: p300

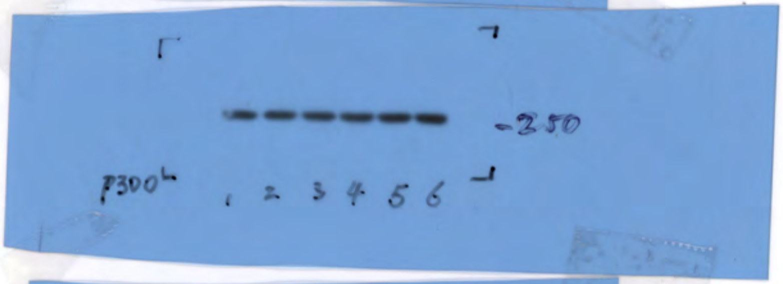


LOAD
IB: α-TUBULIN

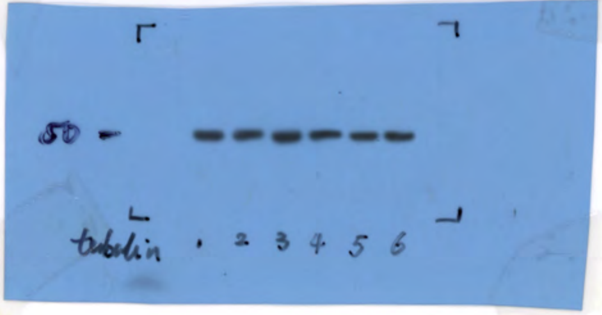

Fig. 3A
(left panel)

HYPOXIA
(HYP)

Acetylation TC - Hyp - endo HIF2α - IP: SP, IB: endo HIF2α, Ack
HCT 116 WHOLE CELL EXTRACTS

IP: HIF-2α
IB: ACETYL-LYSINE

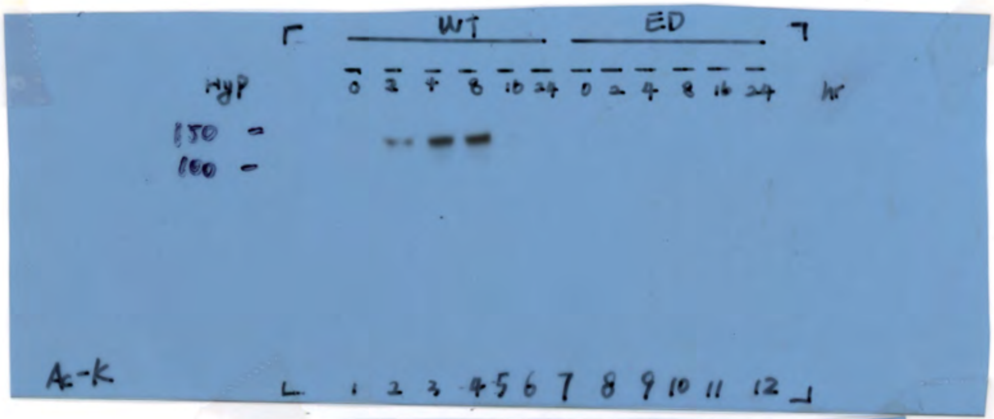


ACSS2 KNOCKDOWN / RESCUE

IP: HIF-2α
IB: HIF-2α

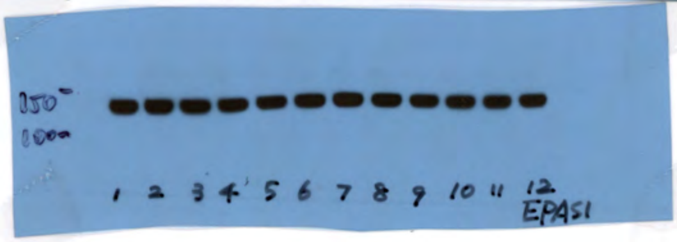


K3
R3

IP: HIF-2α
IB: ACETYL-LYSINE

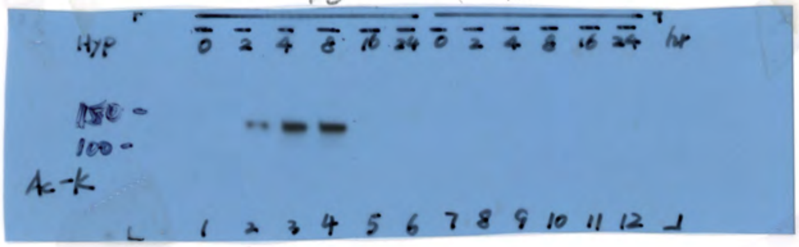


HIF-2α KNOCKDOWN / RESCUE

IP: HIF-2α
IB: HIF-2α

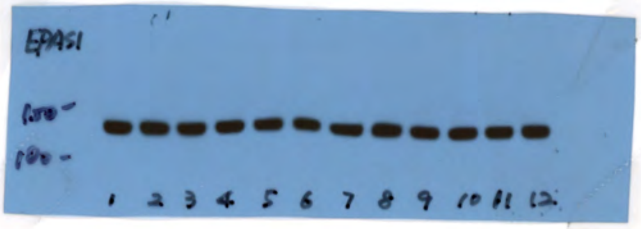

Low Glucose (LG)

Acetylation TC_ LG_ endo HIF2α_ IP: SP, IB: endo HIF2α, Ack in HCT116 WHOLE CELL EXTRACTS

Fig. 3A (right panel)

IP: HIF-2α
IB: ACETYL-LYSINE

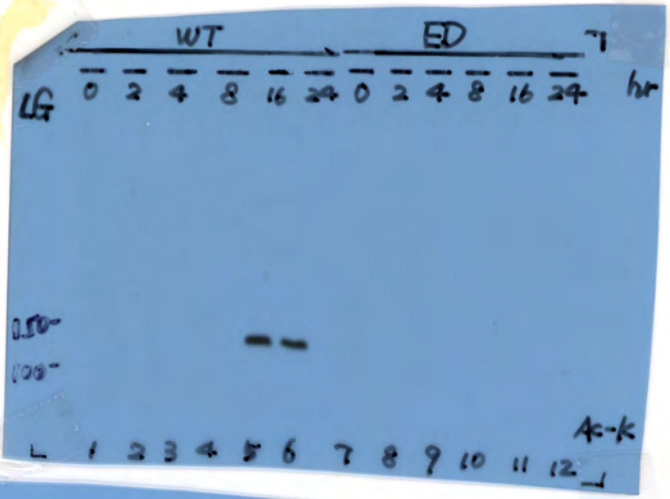


Acss 2 KNOCKDOWN / RESCUE

IP: HIF-2α
IB: HIF-2α

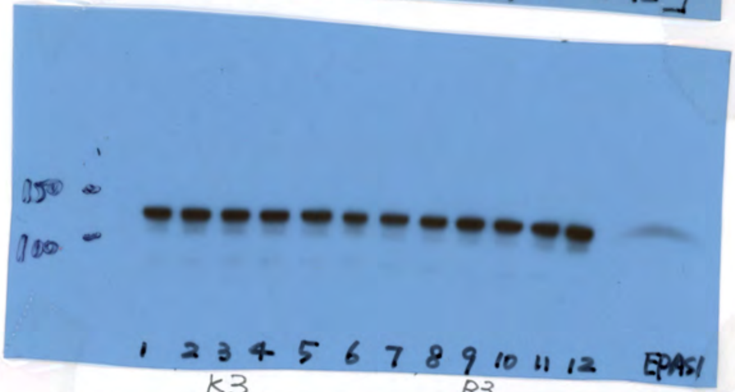


K3 R3

IP: HIF-2α
IB: ACETYL-LYSINE

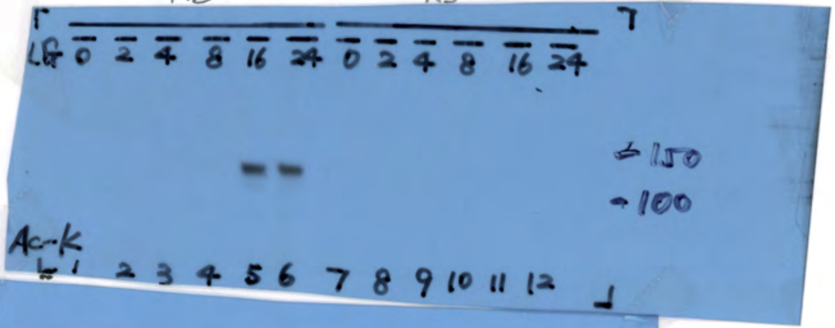


HIF-2α KNOCKDOWN / RESCUE

IP: HIF2α
IB: HIF-2α

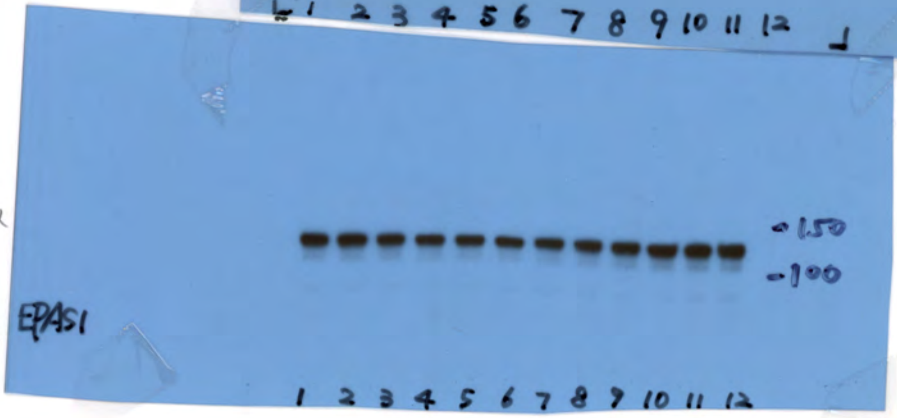

HYPOXIA (HYP)

CoIP_ Normoxia, Hypoxia_ endo HIF2α, cbp, p300_ WT/ED in HCT116 WHOLE CELL EXTRACTS

Fig. 3B (left panel) lower

Acss2 KNOCKDOWN/RESCUE

IP: HIF-2α
IB: CBP

IP: HIF-2α
IB: p300

IP: HIF-2α
IB: HIF-2α

INPUT
IB: α-TUBULIN

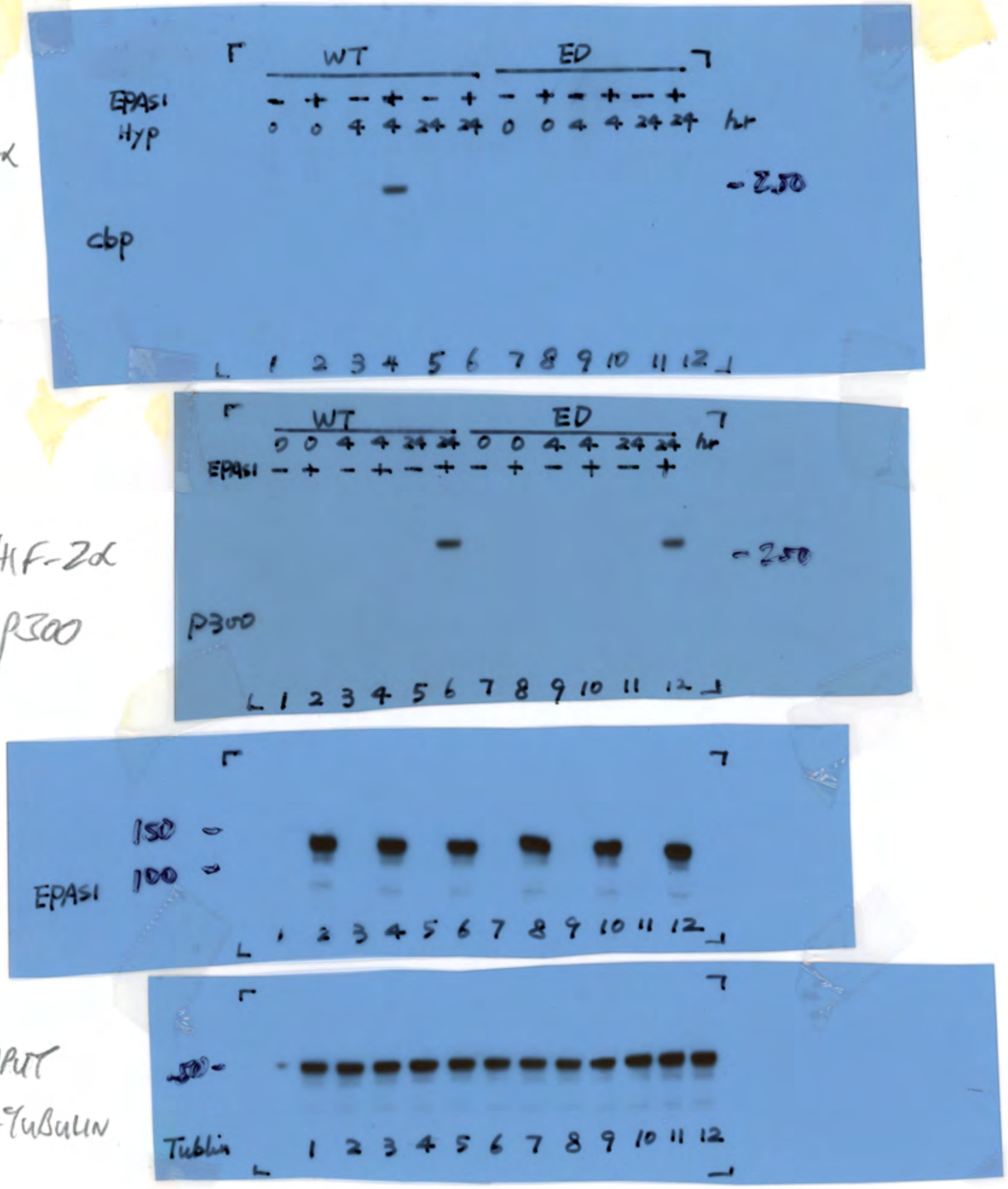

Fig. 3B
(left panel)
upper

HIF-2α
KNOCKDOWN / RESCUE

CoIP_ Normoxia, Hypoxia _ endo HIF2α, cbp, p300
_ K3/R3 in HCT116 WHOLE CELL EXTRACTS

HYPOXIA
(HYP)

IP: HIF-2α
IB: CBP

IP: HIF-2α
IB: p300

IP: HIF-2α
IB: HIF-2α

INPUT
IB: α-TUBULIN

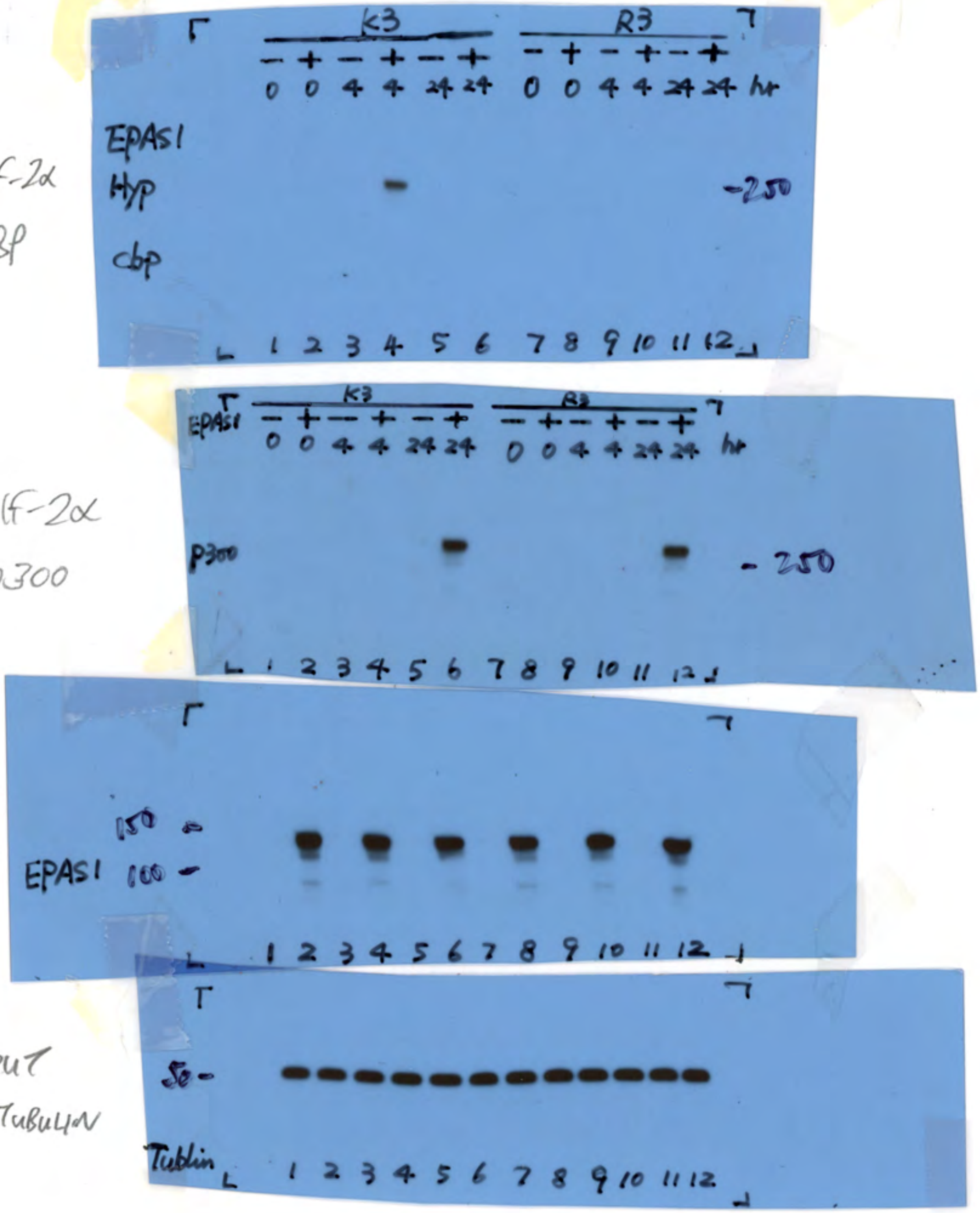

CoIP _ Normoxia, Lowglucose _ endo HIF2α, cbp, p300
WT/ED in HCT116 WHOLE CELL EXTRACTS

Fig 3B (right panel) lower

Low Glucose (LG)

Acss2 KNOCKDOWN / RESCUE

IP: HIF-2α
IB: CBP

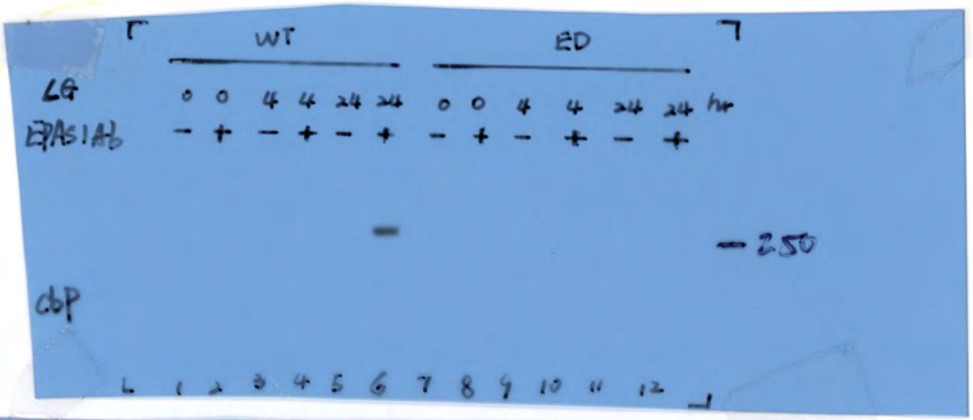


IP: HIF-2α
IB: p300

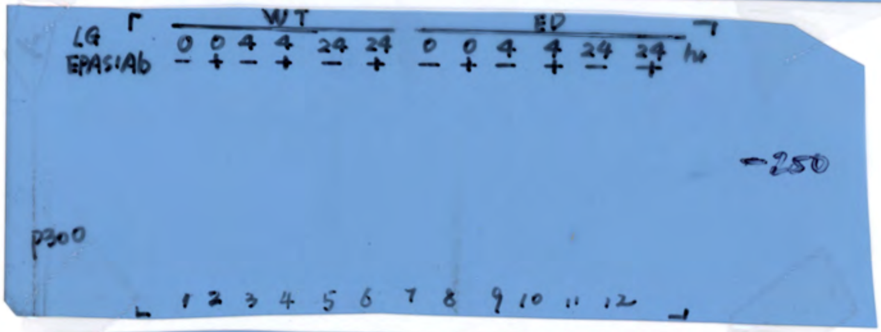


IP: HIF-2α
IB: HIF-2α

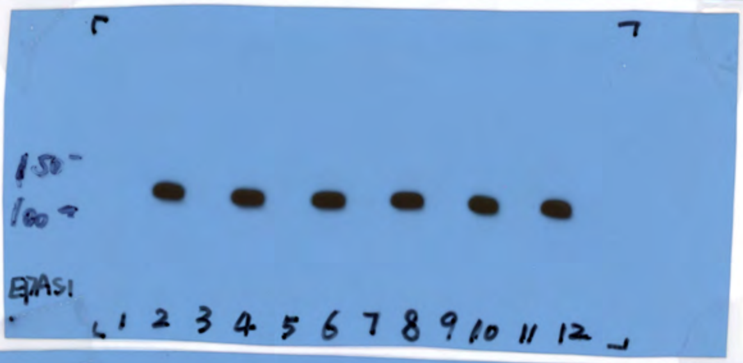


INPUT
IB: α-TUBULIN

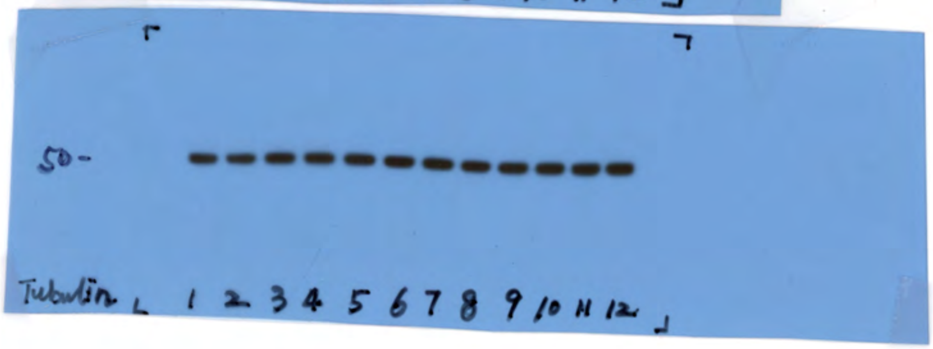

CoIP- Normoxia, Lowglucose - endo HIF2α, cbp, p300.
K3/R3 in HCT116 WHOLE CELL EXTRACTS

Low GLUCOSE (LG)

Fig. 3B (right panel) upper

HIF-2α KNOCKDOWN / RESCUE

IP: HIF-2α
IB: CBP

IP: HIF-2α
IB: p300

IP: HIF-2α
IB: HIF-2α

INPUT
IB: α-TuBulin

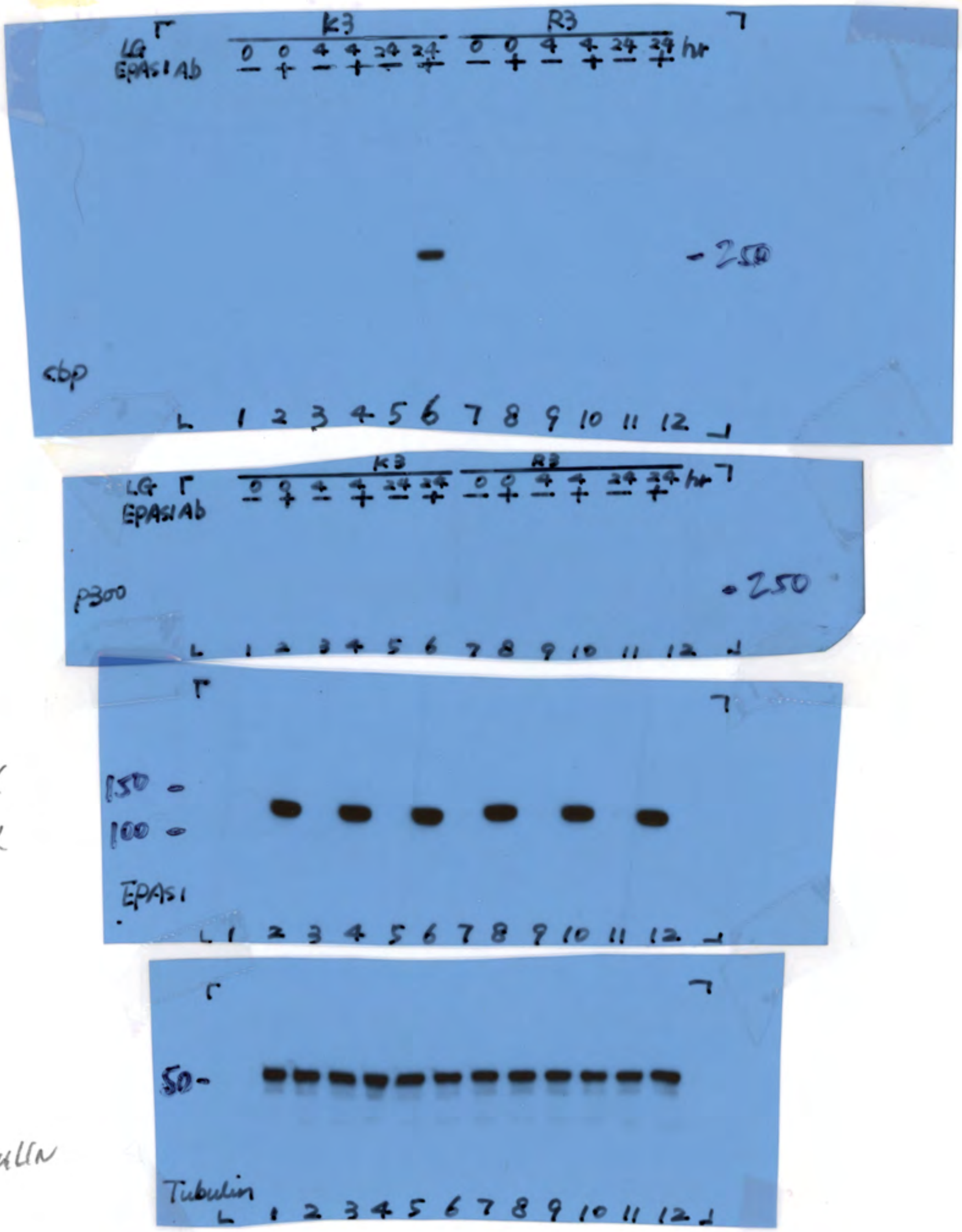

HYPOXIA (HYP)

Acetylation TC_ Hyp_endo HIF2α_ IP; sP, IB: endo HIF-2α, AcK
HT29 WHOLE CELL EXTRACTS

Fig.-4A (left panel)

WT ED

IP: HIF-2α
IB: ACETYL-LYSINE-

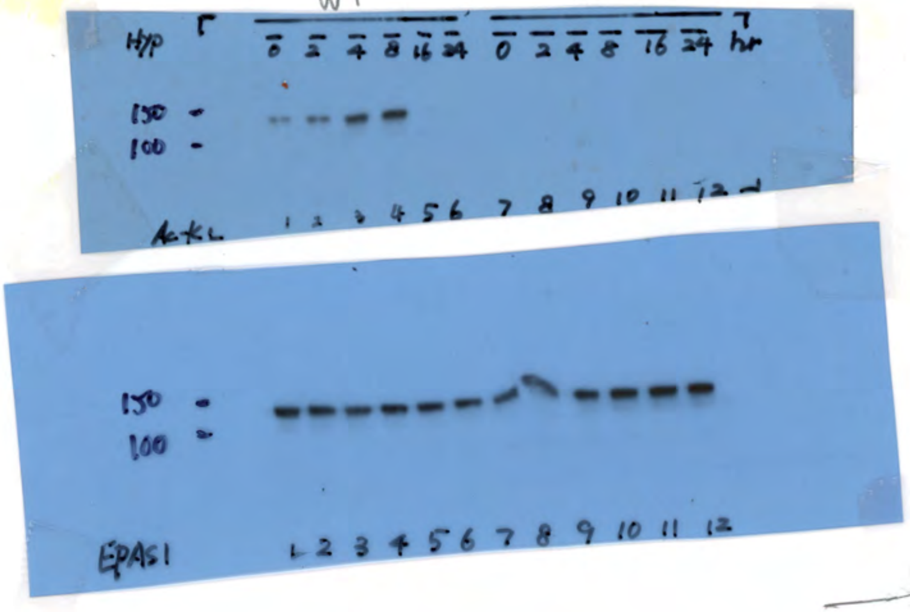


IP = HIF-2α
IB : HIF-2α

Acss2 KNOCKDOWN / RESCUE

K3 R3

IP = HIF-2α
IB : ACETYL-LYSINE

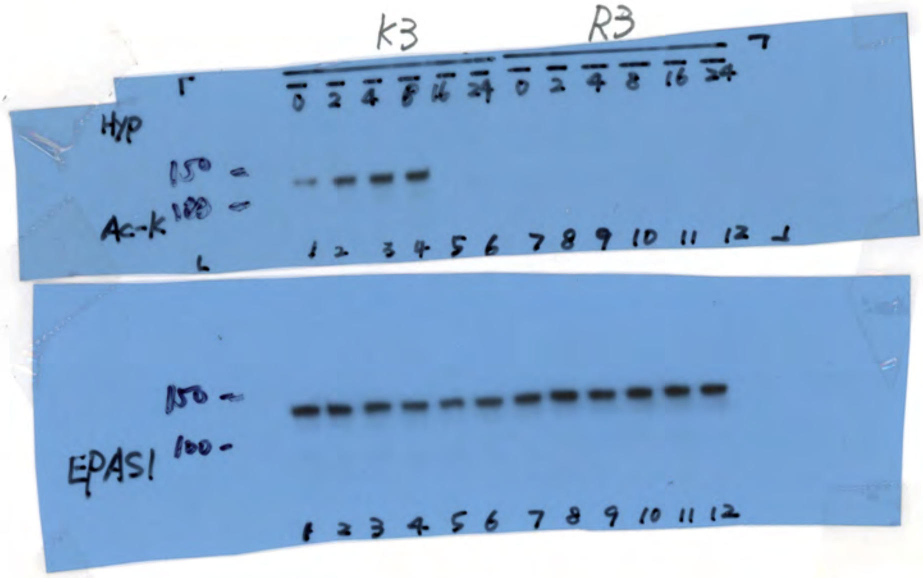


IP: HIF-2α
IB: HIF-2α

HIF-2α KNOCKDOWN / RESCUE

Acetylation TC_ LG_ endo HIF2α_ IP: SP, IB endo HIF2α, AcK
HT29 WHOLE CELL EXTRACTS

Fig 4A
(right panel)

Low Glucose
(LG)

WT ED

IP: HIF-2α
IB: ACETYL-LYSINE

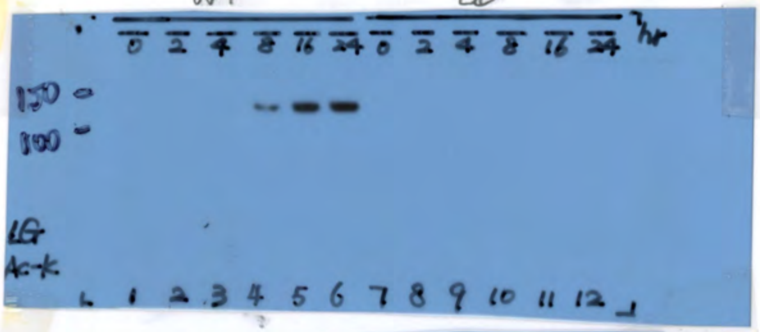


Acss2
KNOCKDOWN/RESCUE

IP= HIF-2α
IB= HIF-2α

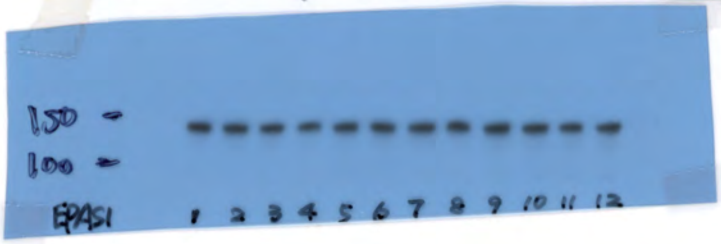


K3 R3

IP: HIF-2α
IB: ACETYL-LYSINE

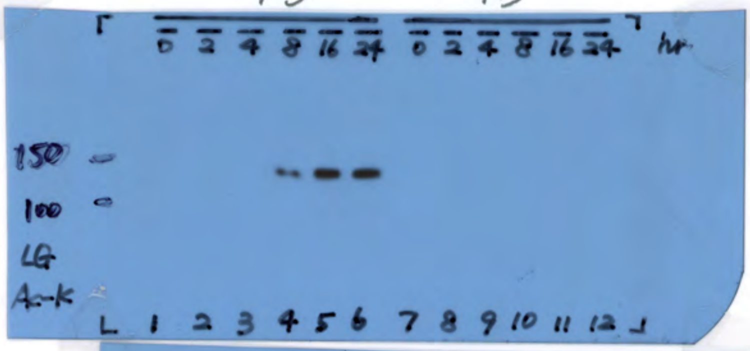


HIF-2α
KNOCKDOWN/RESCUE

IP: HIF-2α
IB - HIF-2α

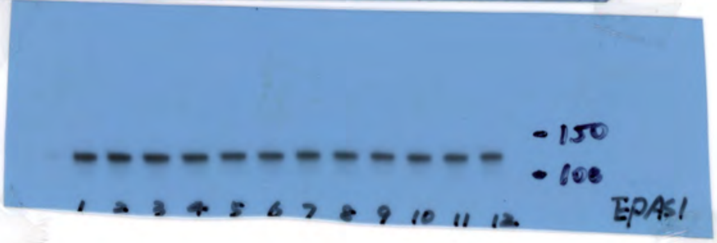

CoIP _ Normoxia, Hypoxia _ endo HIF2α, cbp, p300 _ WT/ED in HT29 WHOLE CELL EXTRACTS

Fig. 4B (left panel) lower

HYPOXIA (HYP)

Acss2 KNOCKDOWN/RESCUE

IP: HIF-2α
IB: CBP

IP: HIF-2α
IB: p300

IP: HIF-2α
IB: HIF-2α

INPUT
IB: α-TUBULIN

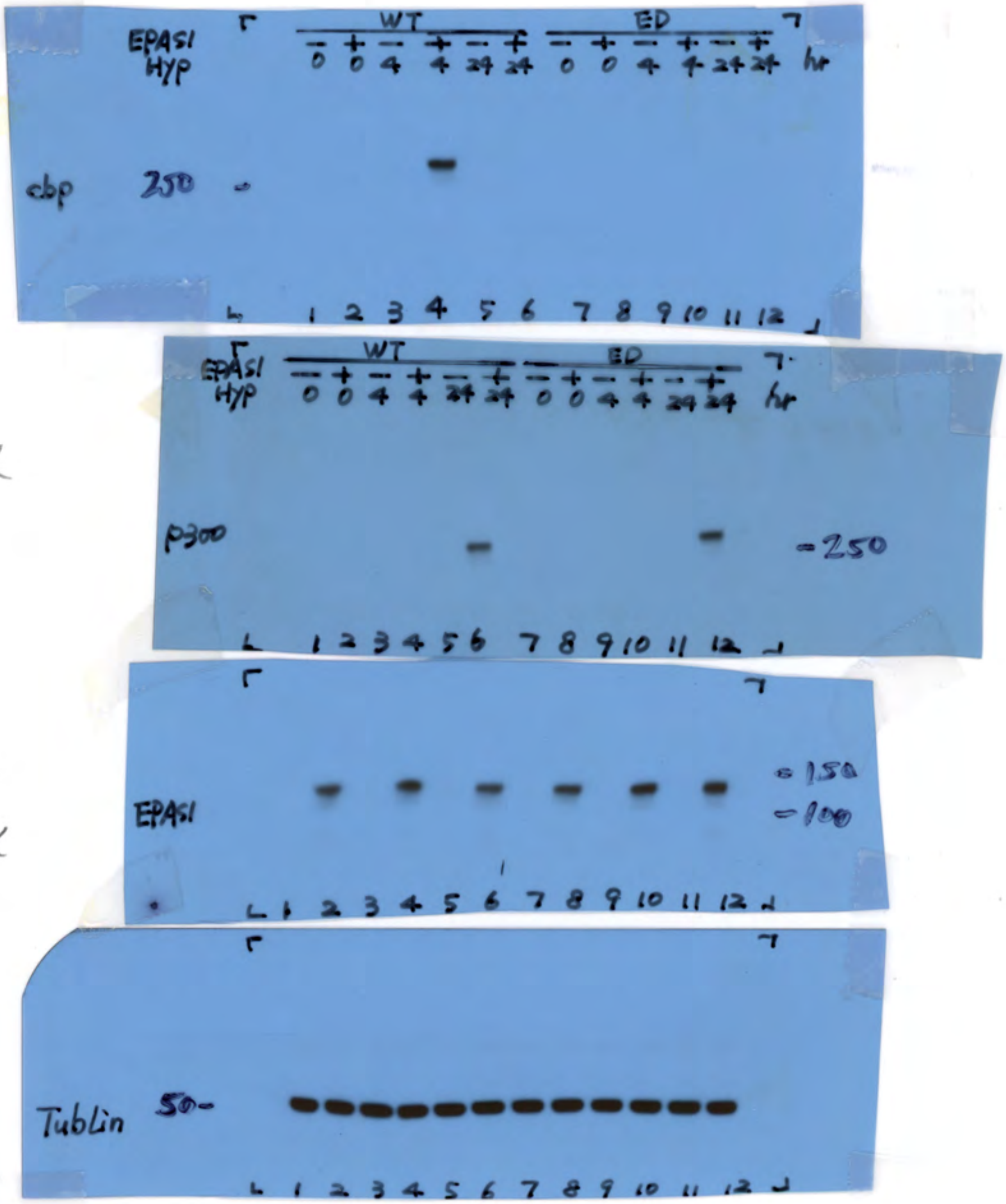

CoIP _ Normoxia, Hypoxia_ endo HIF2α, cbp, p300

_ K3/R3 in HT29 WHOLE CELL EXTRACTS

Fig. 4B

(left panel)
upper

HYPOXIA
(HYP)

HIF-2α

KNOCKDOWN/RESCUE

IP: HIF-2α
IB: CBP

IP: HIF-2α
IB: p300

IP: HIF-2α
IB: HIF-2α

INPUT
IB: α-TUBULIN

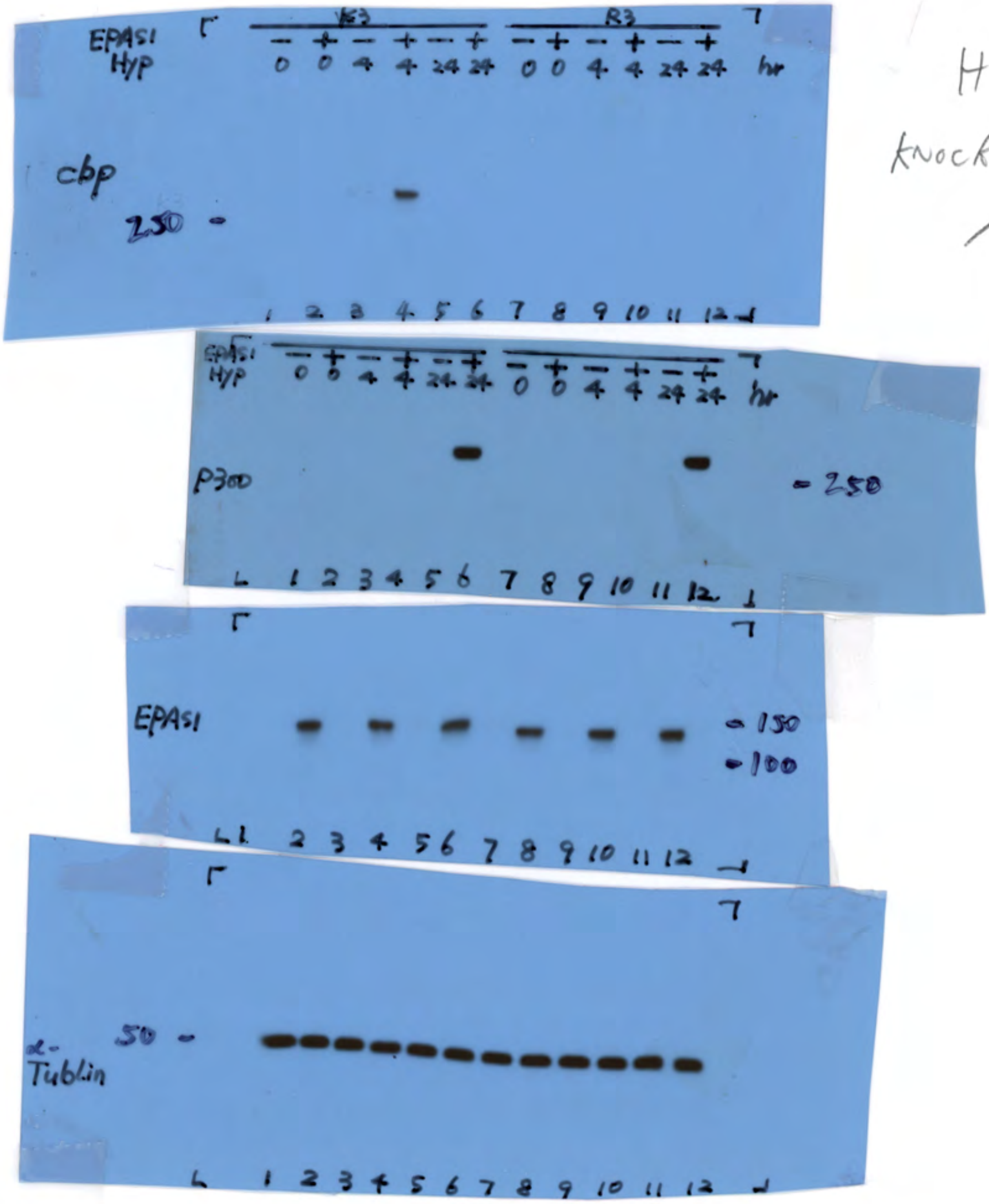

CoIP_ Normoxia, Lowglucose _ endo HIF2α, cbp, p300 _WT/ED in HT29 WHOLE CELL EXTRACTS

Fig. 4B (right panel) lower

Low Glucose (LG)

Accs2 KNOCKDOWN / RESCUE

IP: HIF-2α
IB: CBP

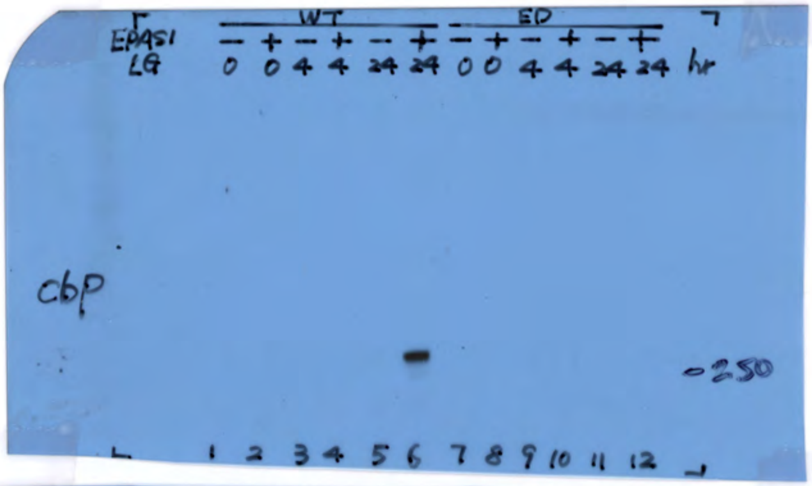


IP: HIF-2α
IB: p300

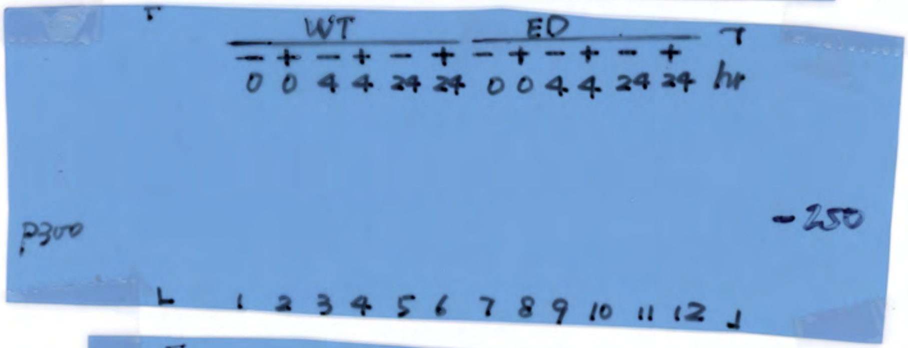


IP: HIF-2α
IB: HIF-2α

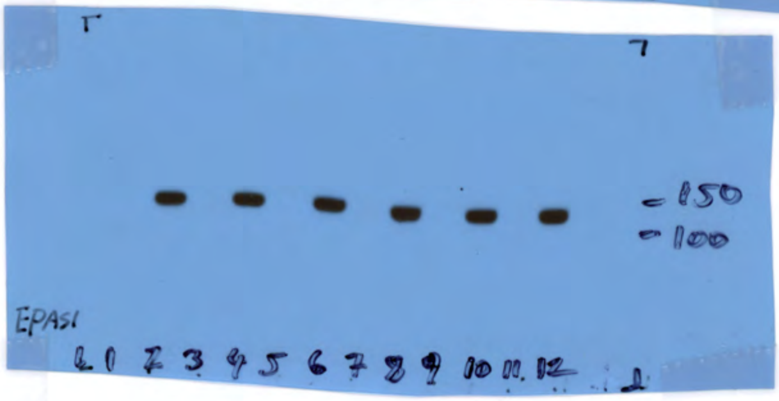


INPUT
IB: α-TUBULIN

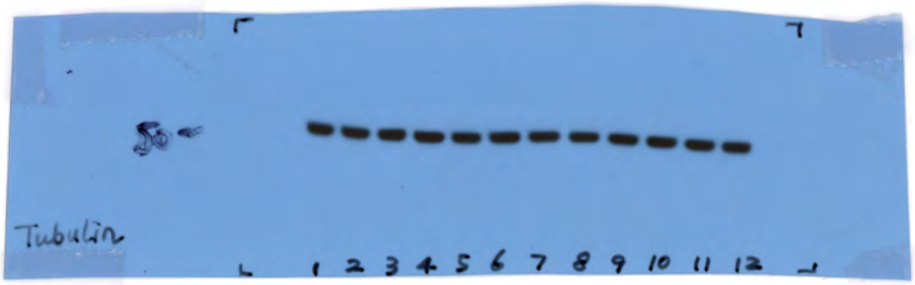

CoIP - Normoxia, low glucose - endo HIF-2α, Cbp, P300
K3/R3 in HT29 WHOLE CELL EXTRACTS

Fig. 4B (right panel) upper

Low Glucose (LG)

HIF-2α KNOCKDOWN/RESCUE

IP: HIF-2α
IB: CBP

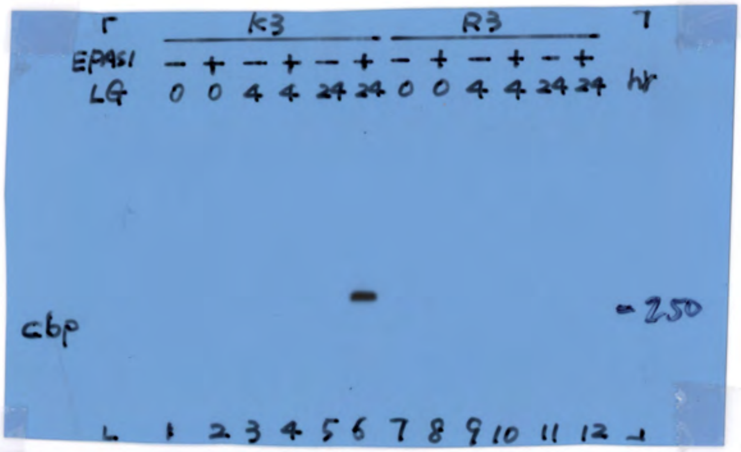


IP: HIF-2α
IB: P300

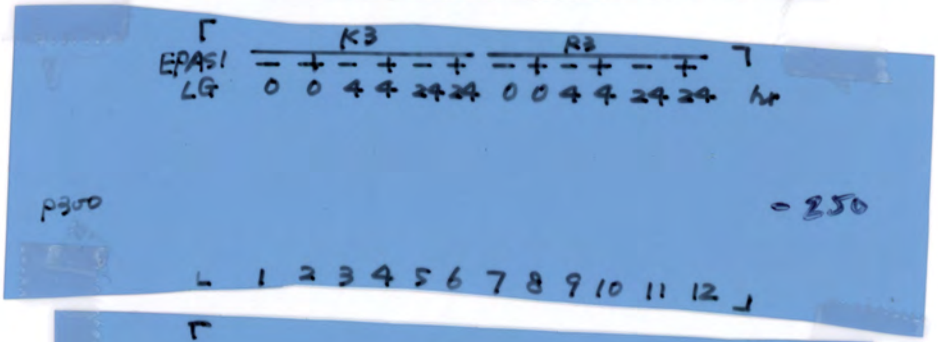


IP: HIF-2α
IB: HIF-2α

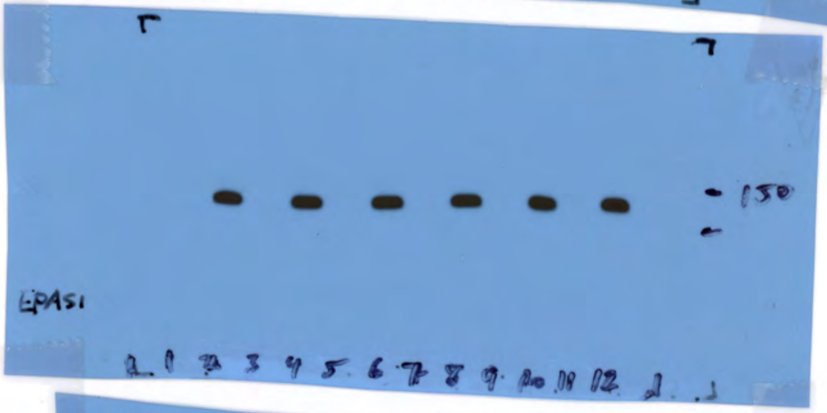


INPUT
IB: α-TUBULIN

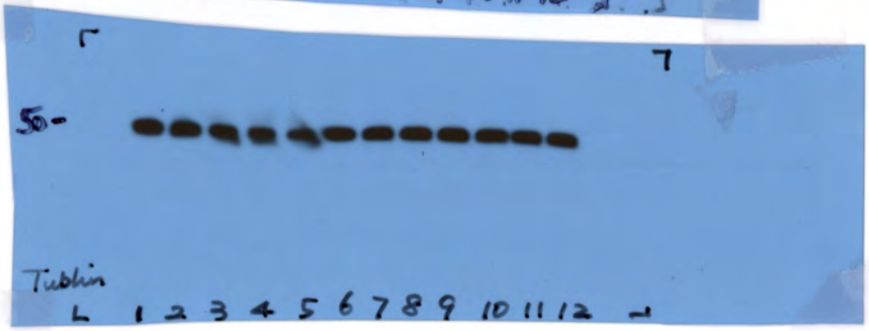

Supplement: S1 Fig — Scans of all films for immunoblots presented in this study. (PDF) [file pone.0282223.s001.pdf]
